# Clonal Hematopoiesis Landscape in Frequent Blood Donors

**DOI:** 10.1182/blood.2024027999

**Published:** 2025-05-22

**Authors:** D. Karpova, H. Huerga Encabo, E. Donato, S. Calderazzo, M Scherer, M. Llorian-Sopena, AM. Leppä, R. Würth, P. Stelmach, D. Papazoglou, A. Ferrelli, S. Ngo, I. Kotova, S. Harenkamp, K. Zimmer, D. Wolf, J. Panten, J. Reed, A. Przybylla, E. Seifried, A. Kopp-Schneider, L. Velten, JF. DiPersio, TN. Wong, D. Bonnet, H. Bonig, A. Trumpp

**Affiliations:** 1 Division of Stem Cells and Cancer, German Cancer Research Center (DKFZ) and DKFZ-ZMBH Alliance, 69120 Heidelberg, Germany; Heidelberg Institute for Stem Cell Technology and Experimental Medicine (HI-STEM gGmbH), and German Cancer Consortium (DKTK), 69120 Heidelberg, Germany; 2 Division of Oncology, Department of Medicine, Washington University School of Medicine, St. Louis, MO; 3 Institute for Transfusion Medicine and Immunohematology, Goethe University Hospital Medical School, German Red Cross Blood Donor Service, Frankfurt, Germany; 4 Hematopoietic Stem Cell Laboratory, The Francis Crick Institute, London, NW1 1AT, UK; 5 Division of Biostatistics, German Cancer Research Center (DKFZ), Im Neuenheimer Feld 581, Heidelberg, 69120 Germany; 6 Computational Biology and Health Genomics, Centre for Genomic Regulation (CRG), Barcelona Institute of Science and Technology (BIST), 08003 Barcelona, Spain; 7 Division of Cancer Epigenomics, German Cancer Research Centre (DKFZ), 69120 Heidelberg, Germany; 8 Bioinformatics and Biostatistics, The Francis Crick Institute, 1 Midland Road, London, NW1 1AT, UK; 9 European Molecular Biology Laboratory, EMBL, Hamburg, Structural Biology, Notkestrasse 85, 22607 Hamburg, Germany; 10 University Hospital Medical School Innsbruck, Internal Medicine V, Department of Hematology and Oncology, Innsbruck, Austria; 11 Division of Regulatory Genomics and Cancer Evolution, German Cancer Research Center (DKFZ) and DKFZ-ZMBH Alliance, 69120 Heidelberg, Germany; 12 Division of Cancer Biology, Department of Medicine, University of Michigan Medical School, Ann Arbor, MI

## Abstract

Blood donation is a life-saving practice, but its long-term effects on donor health and hematopoietic stem cells (HSCs) remain largely under-researched. We conducted a comprehensive analysis of clonal hematopoiesis (CH) in frequent donors (FD; >100 donations) compared to sporadic control donors (CD, <5 donations). Although the overall incidence of CH as well as the clonal size were similar between the groups, we identified a distinct mutational pattern in the DNMT3A gene, the most frequently affected in CH. Unlike HSCs carrying preleukemic DNMT3A R882 mutations, HSCs harboring FD enriched *DNMT3A* variants exhibited balanced lineage output in primary human samples but gained a competitive advantage when stimulated with erythropoietin (EPO), a hormone which increases in response to blood loss. Our findings highlight a nuanced ongoing Darwinian evolution at the somatic stem cell level, with EPO emerging as a novel environmental factor that favors HSCs carrying specific *DNMT3A* mutations.

## Main Text

Access to blood products is the backbone of modern medicine. The burden of blood donation is shouldered by a small group of altruistic healthy volunteers with only ~4% of eligible individuals donating (1). To protect the iron stores of donors from critical depletion, whole blood donation is limited to 4 (women) or 6 (men) units per year (2), each unit representing ~10-15% of a donor’s total blood volume (3). Different environmental stressors such as infection, cytokines, chemotherapy or blood loss can trigger active proliferation of HSCs (5–8) which in turn affects the acquisition and propagation of genetic lesions (9). The expansion of HSC clones (and their progeny) carrying lesions is defined as clonal hematopoiesis (CH) and its prevalence increases with age (10–12). Considering that all red blood cells (RBCs) are replaced three times per year and each donated unit of blood adds 2.5-5% excess erythropoiesis (14), we studied this specific and unconfounded type of systemic stress, that manifests molecularly in form of increased levels of erythropoietin, on the clonal composition of the hematopoietic system. As specific environmental cues can favor the outgrowth of HSCs carrying certain mutations (15–18) (15–26) we hypothesized that repeated large volume phlebotomy may shape the clonal landscape by promoting clones with functionally distinct types of mutations.

### Frequent blood donation shapes mutational landscape of *DNMT3A*

**Fig. 1:**
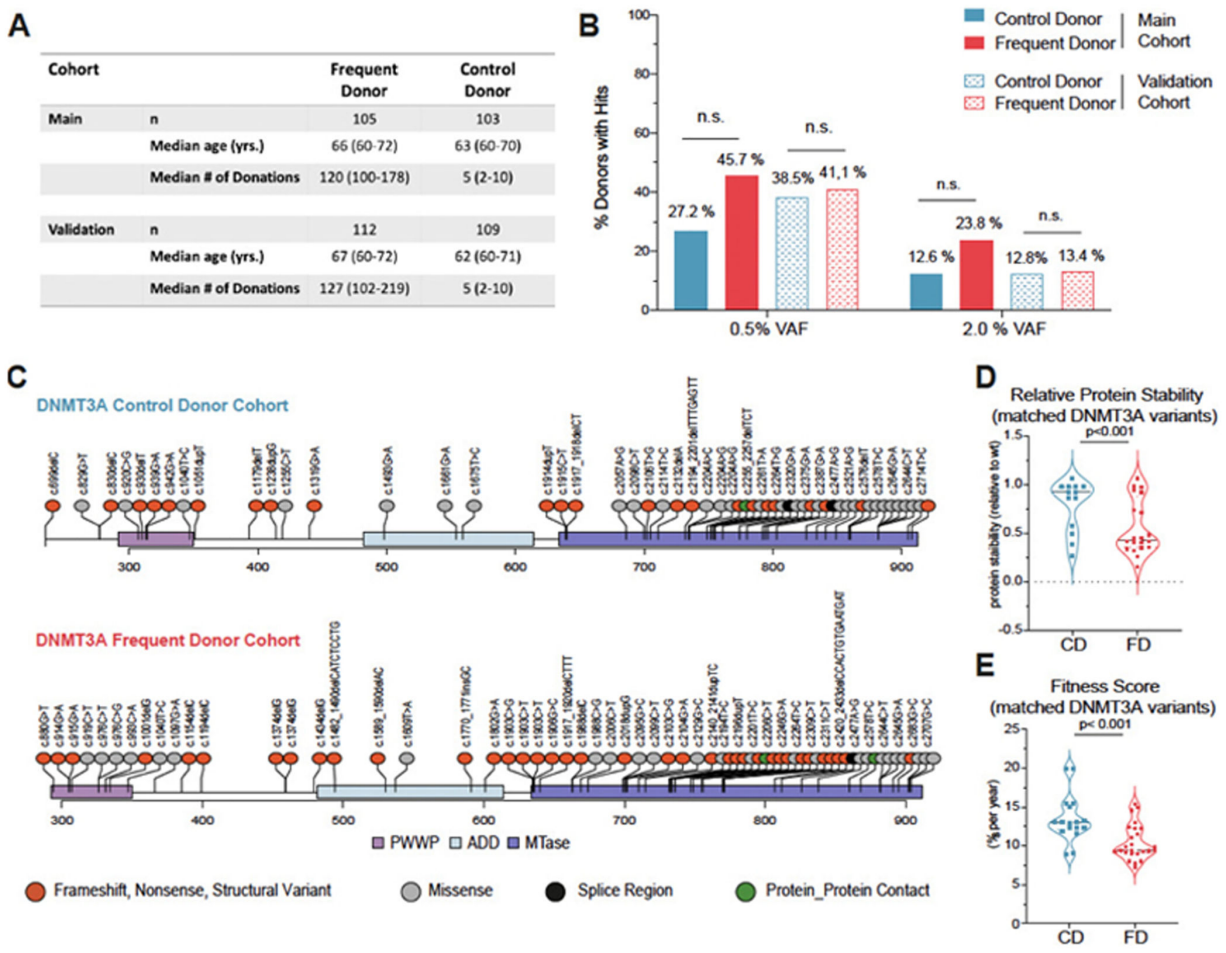
Frequent blood donors show expected CH incidence but distinct *DNMT3A* mutation profile. (**A**) Characteristics of the frequent donor (FD) and control donors (CD) collected and analyzed as the main and validation cohort. (**B**) Percentage of donors with somatic mutations (Hits within the FD and CD cohorts. The cutoff for the VAF (clone size) was set to 0.005 (0.5%). Analysis with a 0.02 (2%) cutoff is shown for comparison. Percentage values are indicated on the bars. Data from the main and validation cohort are plotted separately. For VAF cutoff 0.005, main cohort: adjusted OR/CI: 1.81/0.95-3.49, p=0.074. For VAF cutoff 0.005, validation cohort: adjusted OR/CI: 0.81/0.44-1.48, p=0.49. For VAF cutoff 0.02, main cohort: adjusted OR/CI: 1.33/0.58-3.09, p=0.501. For VAF cutoff 0.02, validation cohort: adjusted OR/CI: 0.72/0.29-1.75, p=0.47. Data from the main and validation cohort are plotted separately. (**C**) LollipopPlot charts with type and location of the mutations in *DNMT3A* shown (detected at a VAF ≥ 0.005, extended FD and main CD). The events are color-coded based on their effects on the protein (see legend). See Supplementary Tables 2a/b and 3a/b for full lists of events. The locations of the PWWP (proline–tryptophan–tryptophan–proline motif), the ADD (ATRX, DNMT3, and DNMT3L)-type zinc finger, and the methyltransferase (MTase) domains are shown. All but three exonic splice region *DNMT3A* mutations (c.2320 G>A, c.2477 A>G in the CD and c.2477 A>G in the FD) are not depicted in the lollipopPlot. Mutations from the (extended) main and validation cohort are plotted together. Fisher test for independence between donor group and mutation class: p=0.404. (**D**) Analysis of stability scores for *DNMT3A* mutations from the FD and CD cohorts that were matched to the variants characterized by Huang et al.^42^ (See Supplementary Table 3a/b), p<0.001. Data from the main and validation cohorts were combined. (**E**) Analysis of the fitness (f) (p<0.001) for *DNMT3A* mutations from the frequent and control donor cohort that were matched to the variants characterized by Watson et al.^33^ (See Supplementary Table 5). Data from the main and validation cohorts are plotted together.

Distinct from most published CH studies (10–12, 27-31) our cohorts consisted exclusively of closely monitored and exceptionally healthy individuals. We first acquired and analyzed a cohort of 105 FD and 103 control donors (CD) individuals (main cohort). Subsequently, a validation cohort of 112 FD and 109 CD individuals was collected using the same criteria as set for the main cohort (Fig. 1A and Supplementary Tables 1a and 1b). No significant difference in the overall CH prevalence between the FD and CD cohorts was found. This was true in both the main and validation cohorts and regardless of the VAF cutoff used, either the sensitive cutoff of 0.5% or the conventional VAF cutoff of 2% (10–12) (Fig. 1B). The VAFs of the detected variants did not significantly differ between the FD and CD (fig. S1A). For all subsequent analyses, the 0.5% VAF was used and the main and validation cohorts were combined. The majority of individuals with CH had a single identified mutation: 64/94 (68.1%) and 46/70 (70.0%) in the CD and FD cohorts, respectively (fig. S1B). Consistent with previous studies (10–12, 32, 33), mutations in *DNMT3A* and *TET2* were the most prevalent in both the FD and CD cohorts (Supplementary Tables 2a/b, 3a/b and 4a/b and fig. S1C), and the VAF distributions of mutations in these two genes did not significantly differ between the cohorts (fig. S1D and Supplementary Tables 3a/3b, 4a/4b). Within the *DNMT3A* gene, mutations were distributed throughout the length of the gene (Fig. 1C), consistent with previous reports(32). The frequency of acute myeloid leukemia (AML) hotspot mutations at position 882 in DNMT3A was low and similar in both cohorts (Supplementary Table 3a/b). Interestingly, within the FD cohort, we observed a trend towards a higher fraction of destabilizing *DNMT3A* variants such as frameshift variants, variants resulting in a premature stop, or structural variants (Fig. 1C). To confirm this, we employed the recently introduced stability score (38) to characterize the *DNMT3A* variants. A total of 20 and 13 *DNMT3A* variants were matched from the FD and CD, respectively. FD *DNMT3A* variants had significantly lower stability scores compared to CD cohort variants (Fig. 1D and Supplementary Table 3a/b). Decreased stability has been directly linked to the regulated degradation of the DNMT3A protein, resulting in a quantitatively reduced enzymatic activity as opposed to a functionally aberrant activity (38). This was further supported by *in silico* structural predictions conducted for a selected set of destabilizing nonsense *DNMT3A* variants from the FD cohort. Three FD *DNMT3A* nonsense variants, W305*, S663fs and E733* were chosen for structural modeling (fig. S1F) in comparison to full length DNMT3A based on their type of mutation and their high VAF (> 5 %). All three mutations were predicted to be degraded due to nonsense-mediated mRNA decay (NMD) (39). Compared to previously described missense mutations capable of interacting with wild-type DNMT3A protein and causing aberrant methylation, including the AML hotspot variant R882H, the three FD variants W305*, S663fs (704*), F733* are predicted solely to cause quantitative reduction in DNA methylation levels (40, 41). Thus, both stability scores and small scale *in silico* structural predictions suggest an enrichment of *DNMT3A* variants with diminished enzyme activity in FD compared to CD. Interestingly, the fitness score (*s-value*) (32) was only 10.5% per year for FD *DNMT3A* variants compared to 13.5% per year for CD *DNMT3A* variants (p<0.001, Fig. 1E), suggesting that the *DNMT3A* variants observed in the FD cohort are less likely to expand during normal aging. Also, the site-specific mutation rate showed a tendency to be lower in the FD cohort (fig. S1E). This suggests that mutations expanding with bleeding associated erythropoietic stress display only moderate fitness in the general population and in the absence of additional stimuli. In line with this, the VAFs of most *DNMT3A* mutations remained stable within short periods of time between two consecutive donations (fig. S1G). For selected donors and *DNMT3A* variants, digital droplet PCR (ddPCR) was performed (fig. S2A) on several mature cell fractions (B-, T- cells and monocytes) along with the immature CD34^+^ compartment. Consistent with previous studies (42–44), all five mutations were detected in all four sorted populations, including T cells, pointing towards their acquisition and selection in multipotent HSCs (fig. S2B).

### *DNMT3A* mutations from frequent donors expand in EPO rich environments but not with inflammatory stimuli

**Fig. 2:**
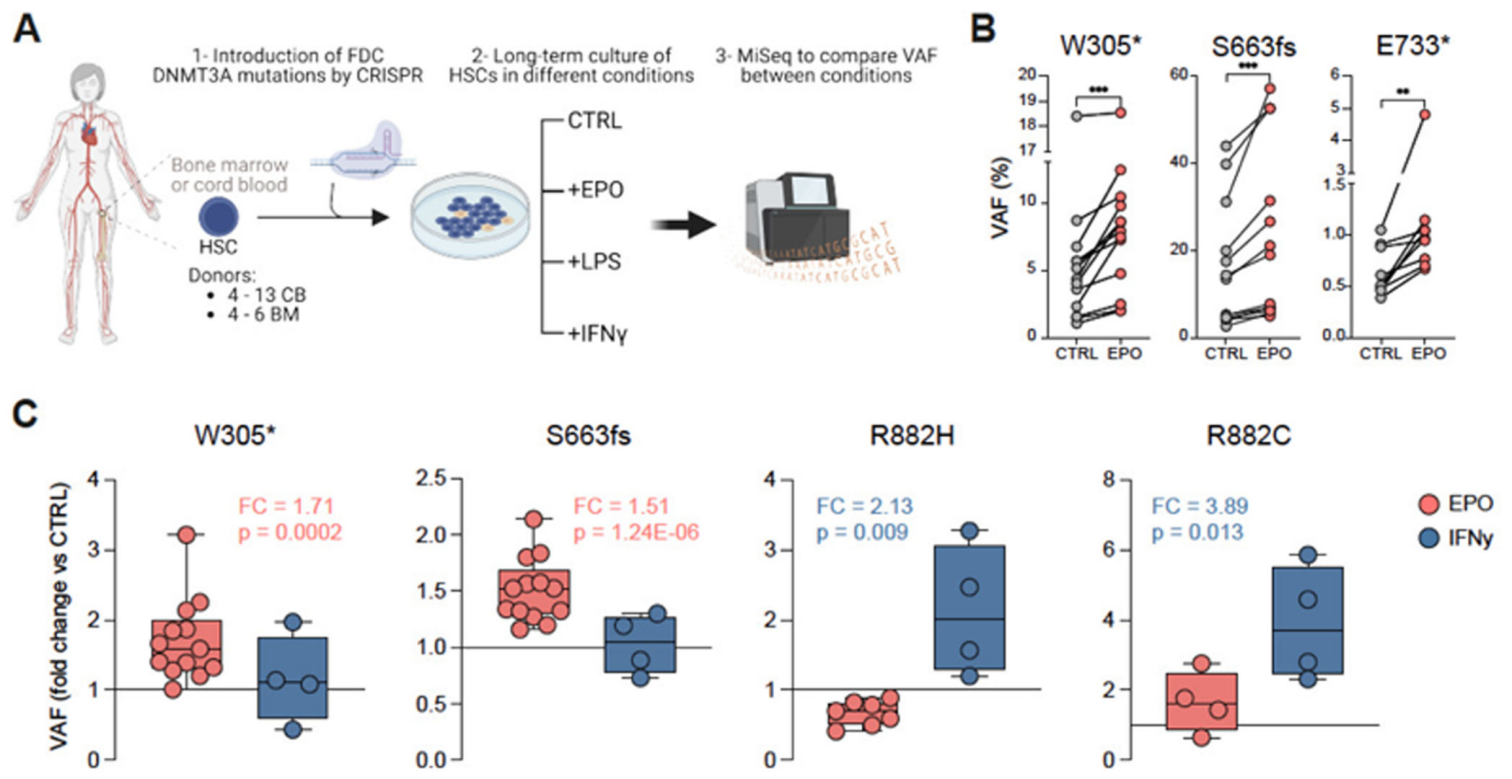
*DNMT3A*-clones associated with blood donation expand in EPO-induced stress while preleukemic R882-mutant clones expand in IFNγ-induced stress. (**A**) Schematic representation of genetic engineering of human HSCs to introduce mutations found in frequent blood donors and perform long term-culture (LTC) in the presence of different stimuli over 4 weeks. VAF between conditions was compared at the end of the co-culture at 4 weeks. (**B**) For each *DNMT3A* mutant clone from FD, a significant increase of the VAF was observed when comparing non-treated (CTRL) and EPO conditions after 4 weeks in culture. Each dot represents an independent biological donor. Paired t-test for each biological donor between different conditions was used for statistical significance. *, p < 0.05; **, p < 0.01; *** p < 0.001. (**C**) Fold change expansion upon different conditions of each mutation in all cord blood donors tested (n = 4-13). For clone S663fs and clone W305* 13 biological donors were tested over 4 independent experiments. For clones R882H and R882C 4-7 biological donors were tested in 2 independent experiments. Each dot represents an independent biological donor. T-test for each biological donor between different conditions was used for statistical significance of the percentage of the *DNMT3A*-mutant clones. *, p < 0.05; **, p < 0.01; *** p < 0.001.

Immediately following whole blood donation which removes 10-12% of the total hemoglobin mass (45), one of the initial responses of the body is to increase the production of erythropoietin (EPO) to stimulate bone marrow erythropoiesis (46). Accordingly, up to two-fold increased concentrations of EPO are detected in the serum of blood donors for up to 56 days following blood donation (46). We next sought to functionally investigate a potential link between the variants found in frequent donors and EPO. The same three *DNMT3A* mutations we had previously subjected to structural analysis (fig. S1F) were also reconstructed and functionally analyzed *in vitro*. Using CRISPF/Cas9, monoallelic W305*, S663fs (704*) and E733* mutations were introduced into primary human HSCs (fig. S3A). *DNMT3A*-edited HSCs were cultured using long-term culture (LTC) assays in the presence or absence of EPO or inflammatory stimuli (IFNγ or LPS) and the VAFs of the introduced *DNMT3A* mutations were assessed after four weeks (Fig. 2A). Of note, we observed that all assessed FD *DNMT3A* mutations expanded in an EPO rich culture (Fig. 2B) which promoted robust erythroid differentiation characterized by an expansion of CD235a^+^CD71^+^ cells (fig. S3B-C) as expected. The 2-fold expansion of FD *DNMT3A* variants with EPO was observed using two different doses of EPO, but IFNγ or LPS failed to expand FD *DNMT3A* variants (fig. S3D-E). Two known preleukemic *DNMT3A* variants, R882C and R882H, were also engineered and similarly analyzed. In sharp contrast with FD *DNMT3A* variants, both R882 clones were highly responsive to IFNγ but unresponsive to EPO (Fig. 2C). Similar observations were made with human bone marrow derived CD34+ cells (fig. S3F). This distinct mutation specific pattern of responsiveness to different environmental cues was also observed when HSPCs harboring FD variants or preleukemic variants were co-cultured together pointing towards cell intrinsic effects (fig. S3G). Thus, different *DNMT3A* variants can introduce a characteristic selective advantage under distinct environmental conditions. Conversely, their presence is suggestive of the environmental signals to which the cells were previously exposed to. Clones with preleukemic mutations tend to be selected in inflammatory environments, while the *DNMT3A* clones enriched in frequent blood donors are selected in EPO-rich environments and fail to respond to inflammatory cues.

### Frequent blood donor W305* mutation causes a shift in DNMT3A transcript abundance and mediates transcriptional programs associated with heme metabolism

**Fig. 3:**
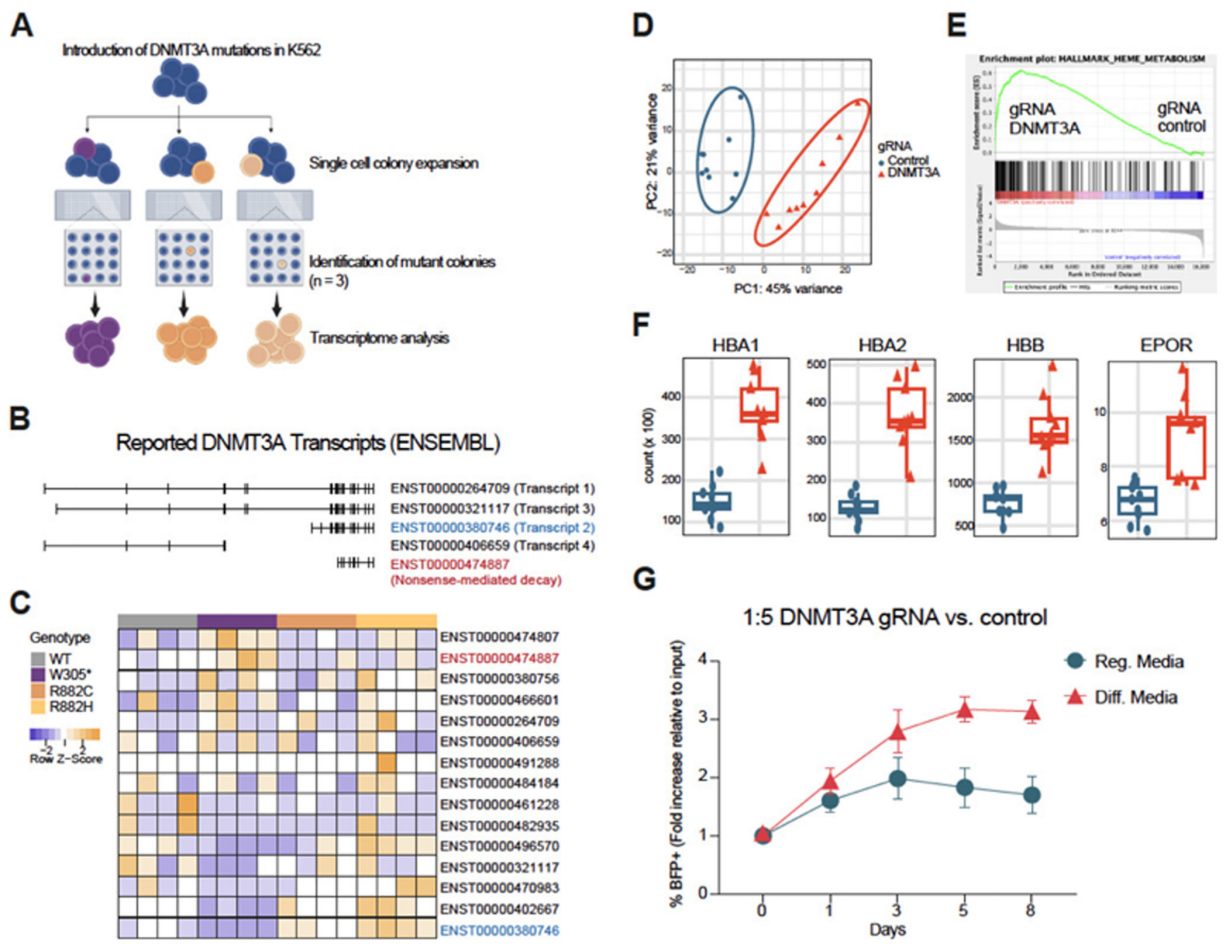
Downregulation of *DNMT3A* associated with W305* FD mutation underlies outgrowth advantage in EPO-rich environments. (**A**) Schematic representation of single cell deposition of K562 after introduction of the mutations by CRISPR, expansion in vitro and colony screening to select the monoclonal colonies harboring specific mutations to perform RNA sequencing. (**B**) Previously identified *DNMT3A* transcripts and the corresponding ENSEMBL annotation. (**C**) Heatmap of *DNMT3A* transcripts annotated in ENSEMBL and detected in the bulk RNASeq. W305* mutant K562 compared to the other genotypes show lower levels of transcripts correlating with previously reported protein-coding transcripts and increased levels of transcripts annotated to undergo nonsense mediated mRNA decay (NMD). (**D**) Principal Component Analysis (PCA) based clustering of transcription profiles of *DNMT3A* downregulated vs. control HUDEP-2 cells. n=9. (**E**) Gene set enrichment analysis (GSEA) of heme metabolism signature in *DNMT3A* downregulated vs. control HUDEP-2 cells. ES, enrichment score; NES, normalized enrichment score; FDR, false discovery rate. (**F**) Normalized expression counts (DESeq2) for indicated genes in *DNMT3A* downregulated (red) vs. control HUDEP-2 (blue) cells. n=9. Padj HBA1, HBA2, HBB and EPOR: 1.39e^-15^, 1.46e^-20^, 1.91e^-19^ and 2.62e^-06^, respectively. (**G**) *DNMT3A* downregulated (BFP+) and HUDEP-2 control (GFP+) cells were co-cultured at the given ratio in regular and erythroid differentiation media. The ratio between BFP and GFP positive cells was analyzed over a period of 8 days and is presented relative to the input. n=3, three independent experiments, measurement in duplicates. P-values Differentiation vs. Regular Media for timepoints Day 1, 3, 5 and 8 were: 0.33, 0.19, 0.03 (*) and 0.01 (*) respectively.

We next sought to understand the molecular mechanisms that could explain the selective outgrowth of FD variants within EPO-rich environments. The W305* variant from the FD cohort was chosen along with preleukemic *DNMT3A* mutations to perform single cell deposition of human erythroleukemic cell line K562 into which these mutations had been previously introduced by CRISPR-Cas9 editing. After colony screening, we selected the monoclonal colonies harboring specific mutations and performed RNA sequencing (Fig. 3A). Pathway enrichment analysis revealed that in W305* mutated clones transcriptional programs associated with heme metabolism were selectively up-regulated (fig. S4A and Supplementary Table 10). Interestingly, when analyzing the differentially expressed genes associated with the W305* mutation, we observed that *DNMT3A* itself was downregulated (fig. S3B and Supplementary Table 11). Considering this observation and based on the different location and predicted changes in the reading frame caused by FD mutations compared to preleukemic R882 mutations (Fig. 1C), we analyzed the abundance of alternative spliced *DNMT3A* transcript isoforms (Fig. 3B). Indeed, W305* mutant K562 cells expressed the protein-encoding transcript 2 at lower levels, while transcripts annotated to undergo nonsense mediated RNA decay (NMD) were highly expressed compared to the WT or R882 *DNMT3A* (Fig. 3C), which is consistent with the *in silico* structural predictions described above. *DNMT3A* transcript 2 was previously reported to be associated with active proliferation and malignancy and we observed that, opposite to the reduced expression associated with W305* variant, R882 variants express higher levels of this *DNMT3A* transcript (Fig. 3C and fig. S4C). To our knowledge, this is the first time distinct effects of different *DNMT3A* mutations on the abundance of different *DNMT3A* transcripts are reported. Our current model explained the EPO-responsiveness of the FD mutants by canonic, yet quantitatively attenuated DNMT3A activity. If this was true, then knock-down of WT DNMT3A should similarly convey EPO-responsiveness, which was therefore tested in the cord blood derived hematopoietic progenitor cell line HUDEP-2. We employed lentivirus based CRISPRi targeting of the *DNMT3A* promoter (fig. S5A). This line has the ability to undergo erythroid differentiation (47, 48). Transcriptomes of CRISPRi-*DNMT3A* targeted HUDEP-2 cells were distinct from controls in which a non-targeting guide RNA was used (Fig. 3D and fig. S5B). More specifically, and consistent with the downstream effects of the W305* mutation in K562 cells, the heme metabolism gene set was enriched upon downregulation of *DNMT3A* in HUDEP-2 cells, while immune response associated genes were depleted (Fig. 3E, fig. S5C and Supplementary Table 12 and 13). Moreover, three hemoglobin genes along with the erythropoietin receptor gene were up-regulated in the *DNMT3A* targeted HUDEP-2 cells (Fig. 3F). Ingenuity pathway analysis identified EPO signaling as the pathway showing the strongest upregulation upon silencing of *DNMT3A* (fig. S5D). This erythroid priming as a result of *DNMT3A* downregulation was functionally confirmed in a competitive culture setting. Of note, under erythroid differentiation conditions, *DNMT3A* downregulated HUDEP-2 cells showed superior growth dynamics (Fig. 3G and fig. S5E), which overall consolidates the notion that FD DNMT3A variants expand in EPO-rich environments.

### Myeloid expansion is associated with R882 mutations but not with EPO responsive DNMT3A variants

**Fig. 4:**
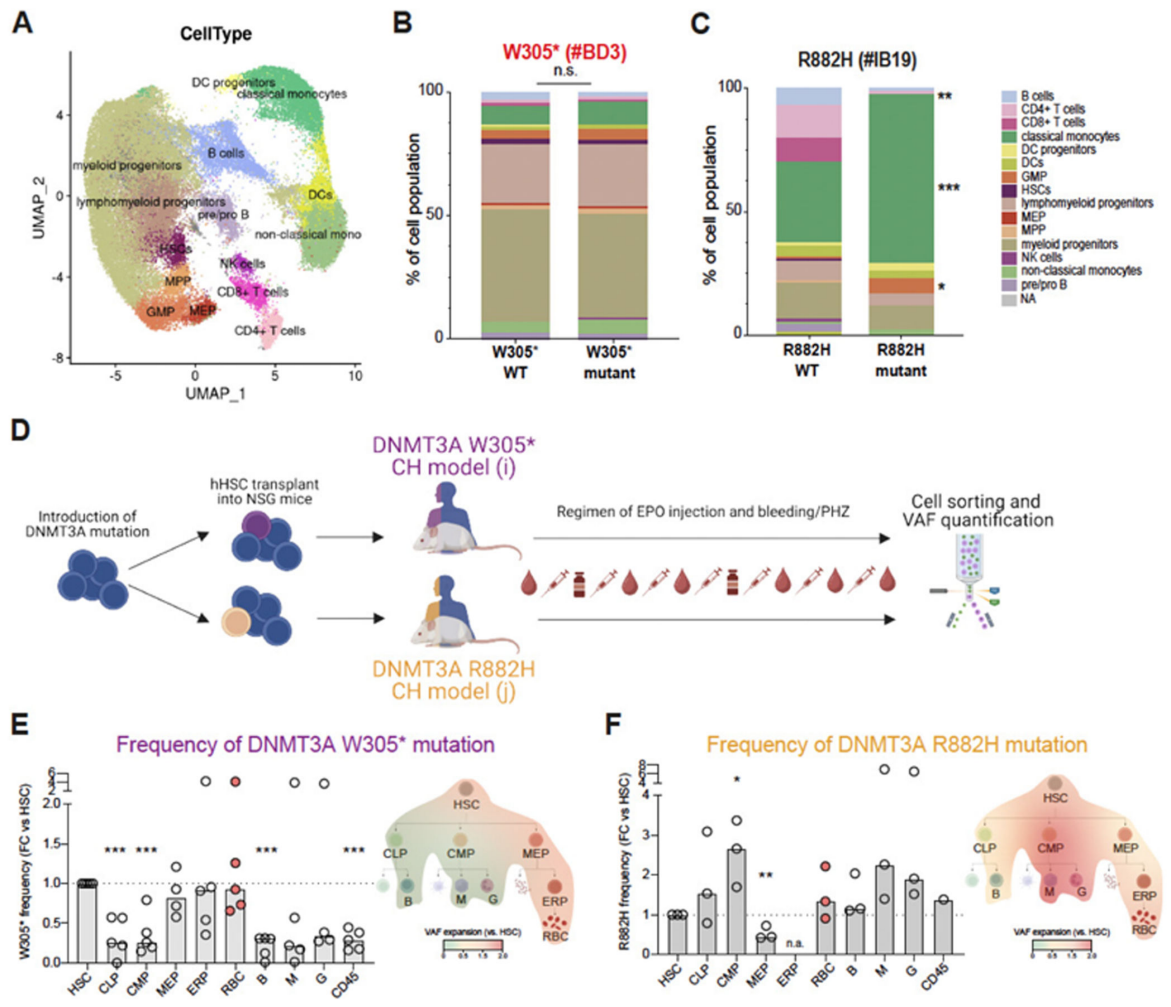
In vivo study of lineage distribution of *DNMT3A* variants. (**A**) UMAP clustering of CD34 enriched samples based on their immunophenotype as defined by expression of 50 unique hematopoietic surface antigens with cell type labels transferred from Triana et al., 2021(51). (**B-C**) Intradonor/Intrapatient, genotype specific cellular composition of indicated donor samples. 15 cell clusters were defined according to the UMAP in **a** as shown in the color-matched legend. Fisher exact test was used for analysis of statistical significance in the contribution of a mutant vs. non-mutant genotype to a given cell population *, p < 0.05; **, p < 0.01; *** p < 0.001. (**D**) Schematic representation of the humanized mice model used to evaluate W305* and R882H behavior within the hematopoietic system after producing sustained erythropoietic stress via successive bleeding/ EPO injection and phenylhydrazine treatment. (**E-F**) Frequency of W305* (**E**) or R882H (**F**) mutations represented as fold expansion from HSPC and mature cell subsets. Each dot represents an individual humanized mouse. t-test was used for statistical significance. *, p < 0.05; **, p < 0.01; *** p < 0.001. Overlaid heatmap representing the fold expansion of each mutation within the different cell populations of the hematopoietic system is provided to visualize the differential lineage bias of the two *DNMT3A* mutations.

To study the impact of different *DNMT3A* mutations in CD34 enriched blood cells of healthy donors, we analyzed these by concurrent assessment of their genotype and the surface marker expression at single cell resolution using the Tapestri platform (49, 50) (fig. S6A). We took advantage of our access to primary samples from blood donors known to carry different *DNMT3A* mutations (Supplementary Tables 3a/b and 4a/b). Two additional samples from hematologically clinically normal individuals, positive for *DNMT3A* R882H CH were included. The full list of samples and corresponding characteristics is shown in Supplementary Table 14. All samples were enriched for CD34^+^ cells prior to surface staining with a 50-antibody panel (Supplementary Table 15b) followed by droplet-based analysis of specific *DNMT3A* mutations in single cells. Projection of the determined cell surface protein expression pattern onto published single-cell proteo-genomic reference maps (51) revealed 15 different cell clusters (Fig. 4A). In the samples from the two frequent donors harboring the variants W305* and E773*, the relative contribution of mutant and WT cells to each of the 15 cell clusters was indistinguishable (Fig. 4B and fig. S6B). In sharp contrast, in all three R882 (2x R882H, 1x R882C) CH samples, a reduction of mature lymphoid cell types (B and T cells) along with an increase in the monocytic fraction was apparent within the mutated compartment compared to the WT cells in the same sample (Fig. 4C, fig. S6C-E and fig. S7A). The *DNMT3A* R366H variant, identified in one of the FD donors and found to be EPO non-responsive, exhibited a similar lineage bias to the R882 variants (fig. S7B). The myeloid bias was furthermore apparent in the granulocyte-monocyte progenitor (GMP) fraction. Here an up to 6-fold higher percentage of cells with a R882H/C or R366H mutation were assigned to the GMP cluster compared to the corresponding WT cells. The numbers of immature hematopoietic stem and progenitor cells in primary samples were generally low, as expected for non-mobilized PB samples, which was particularly the case for R882 donors. To better characterize the effects of the different *DNMT3A* mutations within the stem and progenitor fraction, we next developed a xenograft model. A humanized mouse model engrafted with human HSCs carrying *DNMT3A* W305* or R882H mutations provided additional resolution into the erythroid lineage as well as all the immature populations within the hematopoietic system. To model the environmental pressure of the FD cohort, mice were subjected to a stringent erythropoiesis-inducing regimen of serial bleeding combined with intravascular hemolysis and human EPO injections (Fig. 4D). We then sorted and determined the frequency of each mutation in different stem and progenitor compartments as well as mature lineages (fig. S7C). Consistent with the single cell data from primary patient samples shown above, we observed no significant bias in the presence of the W305* mutation between myeloid (CMP, G, M) and lymphoid (CLP, B) lineages. However, as we gained more resolution, we observed that W305* frequency was underrepresented in myeloid and lymphoid lineages compared to W305* frequency in HSCs as well as within the erythroid lineage (MEP, ERC, RBC) (Fig. 4E). By contrast, R882H mutant DNMT3A cells showed a preferential expansion into the myeloid lineage (CMP, M, G), consistent with previous reports and the lineage bias detected in R882 mutant CH blood samples (Fig. 4F), despite the erythroid stress incurred. Collectively, these data illustrate that the EPO responsive *DNMT3A* W305* variant facilitates stable and balanced blood development during homeostasis, yet it promotes preferential erythroid reconstitution under stress induced by serial blood loss and EPO treatment. This clonal behavior sharply contrasts with the known preleukemic R882 *DNMT3A* mutation, which drives a pronounced myeloid bias both during homeostasis and under erythroid stress.

Our analysis of *DNMT3A* variants at the single cell level in patient samples and reconstitution in humanized mice exposes the distinct impact on lineage contribution from HSC carrying FD *DNMT3A* variants compared to malignant R882 mutation and supports the notion that different stressors have divergent effects on HSCs harboring specific *DNMT3A* mutations. While R882 mutant HSCs display a marked myeloid bias, HSCs with FD *DNMT3A* variants show increased differentiation towards the erythropoietic lineage. Along these lines, single cell transcriptome and methylome analysis from DNMT3A-KO (resembling the effect of FD DNMT3A variants) mice, point towards the expansion and transcriptional bias to an erythro-megakaryocytic fate (42, 52, 53), which is consistent with our *in vitro* data showing FD variants mimicking *DNMT3A* downregulation and competitive outgrowth advantage in EPO rich environments. The increased frequency of EPO-responsive *DNMT3A* variants in frequent donors suggests causality. While the acquisition of a given variant is stochastic, microenvironment-driven evolution in the context of extensive blood donation, a previously undescribed selection pressure, appears to favor this novel class of *DNMT3A* mutations. This phenomenon is an example of the nuanced ongoing Darwinian evolution at the level of a somatic stem cell in healthy individuals. Moreover, in addition to mechanistically defining EPO-responsive DNMT3A mutations, our study introduces an important and uniform reference CH dataset from a novel population of exclusively healthy individuals distinguished by regular exposure to a highly specific systemic stress.

## Supplementary Material

fig. s1

Supplementary Table 1

## References

[1] Ritter S, Hamouda O, Offergeld R (2012). Demography and donation frequencies of blood and plasma donor populations in Germany. Update 2010 and 5-year comparison. Bundesgesundheitsblatt Gesundheitsforsch Gesundheitsschutz.

[2] Association GM Guideline for Manufacturing of Blood and Blood Components and Hemotherapy (Hemotherapy Guidelines).

[3] Nadler SB, Hidalgo JH, Bloch T (1962). Prediction of blood volume in normal human adults. Surgery.

[4] Zhao J, Dahlén T, Brynolf A, Edgren G (2020). Risk of hematological malignancy in blood donors: A nationwide cohort study. Transfusion.

[5] Cabezas-Wallscheid N, Buettner F, Sommerkamp P, Klimmeck D, Ladel L, Thalheimer FB, Pastor-Flores D, Roma LP, Renders S, Zeisberger P, Przybylla A (2017). Vitamin A-Retinoic Acid Signaling Regulates Hematopoietic Stem Cell Dormancy. Cell.

[6] Baldridge MT, King KY, Boles NC, Weksberg DC, Goodell MA (2010). Quiescent haematopoietic stem cells are activated by IFN-gamma in response to chronic infection. Nature.

[7] Essers MA, Offner S, Blanco-Bose WE, Waibler Z, Kalinke U, Duchosal MA, Trumpp A (2009). IFNalpha activates dormant haematopoietic stem cells in vivo. Nature.

[8] Walter D, Lier A, Geiselhart A, Thalheimer FB, Huntscha S, Sobotta MC, Moehrle B, Brocks D, Bayindir I, Kaschutnig P, Muedder K (2015). Exit from dormancy provokes DNA-damage-induced attrition in haematopoietic stem cells. Nature.

[9] Heyde A, Rohde D, McAlpine CS, Zhang S, Hoyer FF, Gerold JM, Cheek D, Iwamoto Y, Schloss MJ, Vandoorne K, Iborra-Egea O (2021). Increased stem cell proliferation in atherosclerosis accelerates clonal hematopoiesis. Cell.

[10] Genovese G, Kahler AK, Handsaker RE, Lindberg J, Rose SA, Bakhoum SF, Chambert K, Mick E, Neale BM, Fromer M, Purcell SM (2014). Clonal hematopoiesis and blood-cancer risk inferred from blood DNA sequence. N Engl J Med.

[11] Jaiswal S, Fontanillas P, Flannick J, Manning A, Grauman PV, Mar BG, Lindsley RC, Mermel CH, Burtt N, Chavez A, Higgins JM (2014). Age-related clonal hematopoiesis associated with adverse outcomes. N Engl J Med.

[12] Xie M, Lu C, Wang J, McLellan MD, Johnson KJ, Wendl MC, McMichael JF, Schmidt HK, Yellapantula V, Miller CA, Ozenberger BA (2014). Age-related mutations associated with clonal hematopoietic expansion and malignancies. Nat Med.

[13] Goodnough LT, Skikne B, Brugnara C (2000). Erythropoietin, iron, and erythropoiesis. Blood.

[14] Al-Huniti NH, Widness JA, Schmidt RL, Veng-Pedersen P (2004). Erythropoietin production rate in phlebotomy-induced acute anemia. Biopharm Drug Dispos.

[15] Hsu JI, Dayaram T, Tovy A, De Braekeleer E, Jeong M, Wang F, Zhang J, Heffernan TP, Gera S, Kovacs JJ, Marszalek JR (2018). PPM1D Mutations Drive Clonal Hematopoiesis in Response to Cytotoxic Chemotherapy. Cell Stem Cell.

[16] Wong TN, Ramsingh G, Young AL, Miller CA, Touma W, Welch JS, Lamprecht TL, Shen D, Hundal J, Fulton RS, Heath S (2015). Role of TP53 mutations in the origin and evolution of therapy-related acute myeloid leukaemia. Nature.

[17] Fabre MA, de Almeida JG, Fiorillo E, Mitchell E, Damaskou A, Rak J, Orru V, Marongiu M, Chapman MS, Vijayabaskar MS, Baxter J (2022). The longitudinal dynamics and natural history of clonal haematopoiesis. Nature.

[18] Tuval A, Shlush LI (2019). Evolutionary trajectory of leukemic clones and its clinical implications. Haematologica.

[19] Hormaechea-Agulla D, Matatall KA, Le DT, Kain B, Long X, Kus P, Jaksik R, Challen GA, Kimmel M, King KY (2021). Chronic infection drives Dnmt3a-loss-of-function clonal hematopoiesis via IFNgamma signaling. Cell Stem Cell.

[20] Cai Z, Kotzin JJ, Ramdas B, Chen S, Nelanuthala S, Palam LR, Pandey R, Mali RS, Liu Y, Kelley MR, Sandusky G (2018). Inhibition of Inflammatory Signaling in Tet2 Mutant Preleukemic Cells Mitigates Stress-Induced Abnormalities and Clonal Hematopoiesis. Cell Stem Cell.

[21] Challen GA, Sun D, Jeong M, Luo M, Jelinek J, Berg JS, Bock C, Vasanthakumar A, Gu H, Xi Y, Liang S (2011). Dnmt3a is essential for hematopoietic stem cell differentiation. Nat Genet.

[22] Kunimoto H, Fukuchi Y, Sakurai M, Sadahira K, Ikeda Y, Okamoto S, Nakajima H (2012). Tet2 disruption leads to enhanced self-renewal and altered differentiation of fetal liver hematopoietic stem cells. Sci Rep.

[23] Wong TN, Miller CA, Jotte MRM, Bagegni N, Baty JD, Schmidt AP, Cashen AF, Duncavage EJ, Helton NM, Fiala M, Fulton RS (2018). Cellular stressors contribute to the expansion of hematopoietic clones of varying leukemic potential. Nat Commun.

[24] Wong TN, Miller CA, Klco JM, Petti A, Demeter R, Helton NM, Li T, Fulton RS, Heath SE, Mardis ER, Westervelt P (2016). Rapid expansion of preexisting nonleukemic hematopoietic clones frequently follows induction therapy for de novo AML. Blood.

[25] Huerga Encabo H, Aramburu IV, Garcia-Albornoz M, Piganeau M, Wood H, Song A, Ferrelli A, Sharma A, Minutti CM, Domart MC, Papazoglou D (2023). Loss of TET2 in human hematopoietic stem cells alters the development and function of neutrophils. Cell Stem Cell.

[26] Shen Q, Zhang Q, Shi Y, Shi Q, Jiang Y, Gu Y, Li Z, Li X, Zhao K, Wang C, Li N (2018). Tet2 promotes pathogen infection-induced myelopoiesis through mRNA oxidation. Nature.

