# Supplementary material for: Clonal Hematopoiesis Landscape in Frequent Blood Donors
Source: Blood. Author manuscript; Available in PMC 2025 Apr 3. (PMC7617549; doi:10.1182/blood.2024027999)
Supplement: fig. s1 [file EMS204243-supplement-fig__s1.pdf]

Supplementary Materials for

Clonal Hematopoiesis Landscape in Frequent Blood Donors

Darja Karpova [1,2,3,4,5]*, Hector Huerga Encabo [6]*, Elisa Donato [1,2,3], Silvia Calderazzo [7], Michael Scherer [8,9], Miriam Llorian-Sopena [10], Aino-Maija Leppä [1,2,3], Roberto Würth [1,2,3], Patrick Stelmach [1,2,3] , Despoina Papazoglou [6], Alessandra Ferrelli [6], Steven Ngo [6], Iuliia Kotova [11], Sabine Harenkamp [5], Kai Zimmer [12,13], Dominik Wolf [12], Jasper Panten [14], John Reed [4], Adriana Przybylla [1,2,3], Torsten Tonn [5], Annette Kopp-Schneider [7], Lars Velten [8], John F. DiPersio[4], Terrence N. Wong [15,#], Dominique Bonnet [6,#,†], Halvard Bonig [5,#, †], Andreas Trumpp [1,2,3,#,†]

Corresponding authors: a.trumpp@dkfz.de; dominique.bonnet@crick.ac.uk; H.Boenig@blutspende.de

The PDF file includes:

Materials and Methods
Figs. S1 to S7
Tables S1 to S15

Materials and Methods

<u>*Human Samples*</u>

Healthy Blood Donors

We performed next-generation sequencing of DNA from peripheral blood (PB) leukocytes of >60-year-old males with an extensive blood donation history (Frequent Donor (FD) Cohort). The control cohort consisted of age-matched donors with few lifetime donations (Control Donor (CD) Cohort). Due to the higher limit of maximum donations per year in males[1], they have the potential for a higher cumulative exposure to erythropoietic stress compared to females. Moreover, variability in menstrual patterns could have complicated the analysis in females. A **total of 217 FD and 212 CD samples** were acquired and analyzed as **cohort 1** and **cohort 2**. Thus, buffy coats, waste products of component preparation from whole blood donations, of selected healthy male volunteer blood donors donating between December 2019 and June 2020 (cohort 1: FD and CD), December 2020 and November 2021 (consecutive samples), as well as May and August 2023 (FD and CD cohort 2) at the German Red Cross Blood Service Baden-Württemberg-Hessen were used for the study. All donors provided written informed consent allowing for anonymous processing of the samples as approved by Ethics Committee vote #329/10. **Cohort 1 samples:** Database query parameters were set to select male individuals over the age of 60 with greater than 100 (FD) or fewer than 10 (CD) whole blood donations. Buffy coats produced in the course of processing of erythrocyte or platelet concentrates meeting these criteria were flagged by the IT system during blood processing. Subsequent verification of the donor characteristics showed that 212 out of 218 samples matched the set criteria during the collection of the cohort 1 samples. Further restriction with regard to inclusion of the donors was related to the quality of the sequencing (coverage depth and number of variants detected per sample) as described under "Analysis of the variants". Based on sequencing metrics 4 additional donors (3x UMI Depth < 400; 1x 225 total variants per sample) were excluded. Thus **105 donors in the FD cohort and 103 donors in the CD cohort**, were included as **cohort 1** (see Fig. 1A and Supplementary Table 1a). These were used for **all statistical comparisons** of the cohort 1 FD and CD. In two of the "wrongly" processed and analyzed samples, from donor #8 (age 53 / 102 donations) and donor #17 (age 64 / 80 donations) mutations in CH drivers *DNMT3A* and *TET2* were identified. Both donors were therefore kept as part of the **extended FD cohort 1** despite being formally too young (donor #8) or having donated too few whole blood units for inclusion (donor #17) since they were of an age where CH can be detected at the set depth of the sequencing and met a general definition of frequent donor. Variants from these extended FD cohort 1 donors were **only considered in qualitative assessment of the variants,** including longitudinal and functional analysis as well as lineage tracing. **Cohort 2 samples:** Database query as well as sequencing quality parameters were set to be exactly the same as for the cohort 1. Moreover, manual curation of the flagged buffy coat bags was performed to verify that the donors met required characteristics. A t**otal of 112 FD and 109 CD samples** were collected (see Fig. 1A and Supplementary Table 1b). Subsequent analysis and variant filtering criteria were set to match those of the first cohort, as described under "Analysis of the variants".

Stroke Patients

Patients were included at the Medical University Innsbruck. Ethics committee of the Medical University Innsbruck, EK-Nr: 1182/2020); Setting of an acute ischemic stroke. For detailed explanation see below under "*Single Cell DNA (scDNA) and immunophenotype analysis of primary samples".*

Sample Processing

Bulk / Whole PBMC Samples

Buffy coats (BC) were kept at RT for up to 3 days prior to shipment and processing at the German Cancer Research Center in Heidelberg. Before initiation of the study, we had verified that short term storage prior to freezing the cell pellet did not affect the leukocyte composition of the samples (see Supplementary Table 7) and therefore did not introduce a bias in case of variable penetrance of certain mutations in different lineages such as have been reported e.g. for certain *DNMT3A* and *TET2* mutations[2]. 5-10 ml BC suspension were washed with PBS and the cell pellet then spun down again to remove excessive plasma prior to shock-freezing at -80 °C. BC cell pellets were thawed on ice immediately prior to DNA isolation.

Selected fractions

For a selection of informative donors, follow-up samples could be collected 1-2 years after the index donation, to assess clonal dynamics. For donors' consecutive sample analysis, BCs were obtained within 24 hours of whole blood donation. Whole PBMC sample was generated and processed as described above. The remaining sample (30-50 ml) was washed and subjected to Ficoll density centrifugation to isolate PBMC for subsequent freezing of live cells. On the days of sorting cryopreserved cells were gently thawed, washed and stained with anti-human CD3, CD14, CD19, CD34 and CD45 antibodies. Sorting of the cell fractions of interest was performed based on following immunophenotypes: T cells: $CD45^{high}$ $CD34^{neg}$ $CD3^{pos}$ $CD14^{neg}$ $CD19^{neg}$, B cells: $CD45^{high}$ $CD34^{neg}$ $CD3^{neg}$ $CD14^{neg}$ $CD19^{pos}$, Monocytes: $CD45^{high}$ $CD34^{neg}$ $CD3^{neg}$ $CD14^{pos}$ $CD19^{neg}$ and HSPC: $CD45^{dim}$ $CD34^{pos}$ $CD3^{neg}$ $CD14^{neg}$ $CD19^{neg}$. Cells were sorted into PBS/BSA, spun down and frozen as pellets at -80 °C until immediately prior to DNA isolation.

DNA isolation

DNA isolated was performed as per manufacturer's instructions using Qiagen DNA isolation kits: QiaAMP DNA Blood Maxi Kit (for up to 1 ml BC cell pellet), QiaAMP DNA Mini kit (for up to 200 µl BC cell pellet and more than 200K sorted cells) and QiaAMP DNA Micro kit (for fewer than 200K sorted cells).

Library preparation and Targeted DNA Sequencing

Library preparation for targeted sequencing of PBMC samples was performed using the Human Myeloid Neoplasms Panel (Qiagen) that covers 141 genes and a total of 436 kilobase pairs. Per sample, 40 ng of genomic DNA were processed according to manufacturer's instructions to obtain dual indexed, molecularly barcoded (unique molecular barcodes, UMI) libraries. Library quality and size were assessed using Agilent 2100 Bioanalyzer. Quantitative verification was performed using qPCR (QIASeq Library Quant Assay Kit, Qiagen) and Qubit dsDNA HS Assay (Life Technologies). Sequencing was performed on an Illumina NextSeq 550 or NovaSeq sequencer, with an average UMI based coverage of 1150x (cohort 1) and 1990x (cohort 2). Raw sequencing data along with the metadata of the analyzed cohorts will be submitted to the European Genome-Phenome Archive (EGA), hosted by the European Bioinformatics Institute and Centre for Genomic Regulation.

Analysis of the variants

Sequencing reads were mapped and annotated using the Qiagen web-based tool for QiaSeq Targeted DNA Enrichment Variant Calling[2]. Read processing pipeline along with the applied variant caller have been

described previously[3] and are available at https://github.com/qiaseq/qiaseq-dna under GNU Affero General Public License v3.0. An average of 170 mutations were called per sample. Full lists of variant calls are shown in Supplementary Tables 2a and 2b and will be submitted to the GEO database. Following criteria were subsequently applied to account for sequencing artefacts as well as to reduce the variant lists to CHIP relevant mutations: Samples with an average UMI depth of less than 400x and an average number of variants per sample higher than 210 were excluded from the analysis. Only variants with a VAF of ≤ 0.4 were extracted to exclude germline variants. VAF values determined by the QiaSeq pipeline were used for all analysis except for the longitudinal sample (donation #2) from donor #444, where the read count ratio extracted from the Integrative Genomic Viewer (IGV, https://software.broadinstitute.org/software/igv/) were used. Synonymous variants, variants predicted to have a low effect along with common SNPs were excluded. The pipeline specific quality parameters "Filter" and "RepRegion" were set to "PASS" and "NA", respectively. Furthermore, given the size of the cohort, variants (same gene and position) found to occur more than 10 times were considered to be panel artefacts and excluded from the final analysis. Moreover, final manual curation of the variants was performed to exclude non-canonical, non-hot spot variants (e.g. MYC c.154_156delCAG) present less than 10 times yet too frequent to not be considered a panel artifact. Lastly, only mutations matched to a reference list of 71 previously curated and validated CH variants[4] were included in the final lists to avoid inclusion of artefacts. Cohort 1 variants included in the final list of mutations had a VAF between 0.0056 and 0.305 and variant allele coverage of an average of 30 UMI family-based reads. Cohort 2 variants included in the final list of mutations had a VAF between 0.0046 and 0.2606 and variant allele coverage of an average of 37 UMI family-based reads. Analyses were run in R software, v 4.0.1. COSMIC database[5] was used for manual curation of the variants.

Lollipop plots for DNMT3A were generated using the lolliplot function from the R package trackViewer. Spliceosome mutations were excluded. Protein domain annotations for the plots were downloaded from https://genome.ucsc.edu/.

Digital Droplet PCR (ddPCR)

Digital droplet PCR was performed for validation of the targeted sequencing as well as when screening for presence of selected mutations in specific cell fractions of the sample. All assays were designed and purchased from Bio-Rad. ddPCR Supermix for Probes (No dUTP, Bio-Rad) was used for all reactions. Assay IDs are listed in the Supplementary Table 8. Reactions were set up according to manufacturer's instructions with 5-50 ng genomic DNA as input and annealing / extension temperatures of 53-55 °C for 40 cycles. Bio-Rad QX200 Droplet Digital PCR System was used for droplet generation and analysis of the samples.

Human HSCs and genome editing of DNMT3A

Umbilical Cord Blood (UCB) was obtained from full-term donors with parental informed consent at the Royal London Hospital (London, U.K.) under approval by the East London Ethical Research committee. Mononuclear cells (MNCs) were isolated by density centrifugation using Ficoll-Paque (GE 67 Healthcare). Anonymized human BM samples from consenting healthy volunteers were provided as approved by Ethics Committee of Goethe University Frankfurt (#329/10). MNCs were depleted for lineage positive cells using

an EasySep Human Progenitor Cell Enrichment Kit (Stem Cell Technologies) and HSPCs isolated as described previously[6]. Lineage$^-$CD34$^+$CD38$^-$ cells were cultured in StemSpanSFEM (Stem Cell Technologies) with 100 ng/mL rhFLT-3L, 100 ng/mL rhSCF, and 100 ng/mL rhTPO for 48 hours. CRISPR editing was then performed with the NEON Transfection system (Thermo Fisher) to introduce the RNP complex using the indicated small guide RNAs and donor templates (Supplementary Table 9). For LTC assays, a MS-5 feeder-layer was seeded at $2x10^4$ cells/cm^2 in a 12-well dish and irradiated (7.5 Gy) after 24 h. 6-12 h later, media was replaced with 1 ml of Myelocult H5100 (Stem Cell Technologies). 48 h following CRISPR-induced modification, 1,000 HSPCs were transferred to MS-5 plates. Once a week, 500 µL of media was replaced with the following stimuli: Erythropoietin (Peprotech; 3 or 15 U/mL as indicated), human IFN-γ (Bio-Techne; 100 ng/mL) or LPS (Sigma; 1 µg/mL). After 4 weeks, cells were collected and analyzed by flow cytometry. Different cell populations were sorted for DNA extraction.

Generation of humanized mice reconstituted with edited hHSCs harboring DNMT3A W305 or R882H mutations.*

NBSGW (NOD/SCID/ IL2rγ−/−/Tyr+/Kit W41J) mice were used in this experiment. Mice are bred in isolators with aseptic standard operating procedures in the Biological Research Facility of The Francis Crick Institute. Once weaned, mice were kept in ventilated cages. All animal experiments were performed under the U.K. Home Office project license (70/8904) in accordance with The Francis Crick Institute animal ethics committee guidance. NBSGW mice aged between 8 – 12 weeks received i.v. injections of hHSC (10,000-20,000 Lin-CD34$^+$CD38$^-$ cells/mouse). Engraftment of the reconstituted human hematopoietic system was validated for each mouse by bone marrow aspiration at 6 weeks before starting the regimen of erythropoietic stress consisting of bleeding by tail vein puncture (0.1 ml) every week combined with, 48 hours after each bleeding, injections of human EPO (50 U/mouse). At week 2 and week 4 of the regimen intraperitoneal injections of phenylhydrazine (20 µg/g) (Sigma Aldrich) were administered instead of bleeding stress. After 10 weeks of treatment the mice were sacrificed 48 hours after the last human EPO injection, after a total of 4 months post-transplantation of hHSCs.

Flow cytometry analysis and cell sorting

Experiments were analyzed at the Flow Cytometry core facility of The Francis Crick Institute using the LSR FORTESSA (BD Biosciences, Heidelberg, Germany) equipped with a 488-nm laser, a 561-nm laser, a 633-nm laser, and a 405-nm laser. For sorting, cell suspensions were filtered through a 35-µm nylon mesh (Corning, Berlin, Germany) and sorted in a BD FACS FUSION cell sorter equipped with 488-nm, 561-nm, 633-nm, and 405-nm lasers. The antibodies used for *in vitro* experiments were: CD45-FITC (clone HI30, Biolegend, San Diego, CA), CD34-PerCP-Cy5.5 (clone 8G12, BD Pharmingen, Heidelberg, Germany) and CD38-PECy7 (clone HIT2, BD Pharmingen) for sorting of HSPCs and CD33-PE (clone P67.6, Biolegend), CD19-APCCy7 (clone HIB19, Biolegend), CD71-APC (clone OKT9, eBioscience, San Diego, CA) and CD235a-FITC (clone HIR2, BD Pharmingen) for flow cytometry analysis. For in vivo experiments two antibody panels were used (see **Supplementary Fig. 7C**). For sorting of mature cells: CD45-PerCP-Cy5.5 (clone HI30, BD Pharmingen), CD235a-PeCy7 (clone GA-R2/HIR2, BD Pharmingen), CD71-APC (clone OKT9, eBioscience), CD19-FITC (clone HIB19, BD Pharmingen), CD14-BV785 (clone 63-D3, Biolegend), CD16-APCCy7 (clone 3G8, Biolegend), CD33-PE (clone WM53, BD Pharmingen). For sorting of stem and progenitor cells: Lineage cocktail-eFluor450 (Invitrogen), CD34-PerCP-Cy5.5 (clone 8G12, BD Pharmingen),

CD38-APCeFluor780 (clone HIT2, eBioscience), CD71-APC (clone OKT9, eBioscience), CD117-PECy7 (clone 104D2, eBioscience), CD45RA-BV786 (clone 5H9, BD Bioscience), CD10-FITC (clone HI10a, BD) and CD123-PE (clone 9F5, BD Pharmingen). Dead cells were excluded by staining with the fluorescent dye DAPI (1 µg/ml; BD Biosciences, Cat# 564907) and gating out the positive cells. All experiments were analyzed with FACSDiva 6.2 (BD Biosciences) and FCS Express 7 software.

DNA sequencing for quantification of DNMT3A variants generated in vitro
Sorted cells or CFUs were pelleted and DNA was extracted using EZNA Tissue DNA kit (Omega Bio-tek, Norcross, GA). Targeted sequencing to the DNMT3A region of interest was performed after PCR amplification using the corresponding primers listed in the Supplementary Table 9.

In silico structural analysis
Models were generated using homology modelling on SWISS-MODE[7,8] based on the crystal structure of DNMT3A available on PDB under the alias 5YX2[9]. UCSF ChimeraX[10] and PyMOL[11] were used for model visualization.

Transcriptome analysis of DNMT3A mutations in a clone using K562
K562 cell line was obtained from the Francis Crick Institute cell service facility. The different DNMT3A mutations were introduced by CRISPR following the same protocol done in HSPCs. In order to isolate and expand clones harboring each mutation we used Dispencell (SEED Bioscience, Epalinges, Switzerland) to seed single cells in 96-well plates. After 1 week we pick and screen for pure colonies carrying each mutation individually. Three independent colonies for each mutation were selected and were expanded for 4 weeks before performing bulk RNASeq. K562 mutant clones were pelleted and RNA was extracted using RNeasy Mini Kit (Qiagen, Hilden, Germany). Total RNA quality was verified in an Agilent 2100 Bioanalyzer (Agilent Technologies, Sta. Clara, CA) and samples with RNA integrity number (RIN) of 8 or above were used prior to library preparation. Libraries were prepared using KAPA Stranded with RiboErase RNA-seq kit (according to the manufacturer's instructions). Briefly, 17–25 ng of starting RNA were subjected first to cytoplasmic and mitochondrial ribosomal RNA (rRNA) depletion by hybridization of complementary DNA oligonucleotides, followed by treatment with RNase H and DNase to remove rRNA duplexed to DNA and original DNA oligonucleotides. Samples depleted of rRNA were then subjected to 94 °C for 6 min in the 2×Fragment, Prime, and Elute Buffer in order to obtain 200–300 bp fragments. cDNA synthesis was run in two steps following the manufacturer's instructions. The ligation step consisted of a final volume of 110 µl of the adaptor ligation reaction mixture with 60 µl of input cDNA, 5µl of diluted adaptor, and 45 µl of ligation mix (50 µl of ligation buffer + 10 µl of DNA ligase). The Kapa Dual- Indexed Adaptors stock concentration was diluted to 1.5mM to get the optimal adaptor concentration for library construction. The ligation cycle was run according to the manufacturer's instructions. To remove short fragments such as adaptor dimers, 2X AMPure XP bead clean-ups were done (0.63 SPRI and 0.7 SPRI). To amplify the library, 15 PCR cycles were applied to the cDNA KAPA mix. Amplified libraries were purified using AMPure XP. The quality and fragment size distributions of the purified libraries were assessed with D1000 ScreenTape assay and reagents using Tapestation 42000 systems (Agilent Technologies). Sequencing was then performed in a NovaSeq 6000 Sequencing System (Illumina, San Diego, CA) with 25

million single-end 100 bp reads/sample. Obtained FASTQ files were uploaded to the R environment where adapter and quality trimming was performed. Read QC and alignment was carried out with nf-core/rnaseq version 3.7, using the following extra parameters "-profile crick --aligner star_rsem". The aligner selected was STAR[12] and read quantification was performed with RSEM[13]. Reads were aligned against the Human genome, using Ensembl GRCh38, release 95. Differential expression analysis was performed at the gene level using DESeq2 (v.1.34.0) within R (version 4.1.1) programming environment. Genes are designated as differentially expressed if Padj < 0.05. Initially, differential expression analysis was done based on the model "0 + Genotype*Treatment + Batch" comparison. Subsequently, in order to find genes different between W305* and the rest, we used the same model as above, but this time all genotypes that were not W305* were considered as part of the same group. Gene Set Enrichment analysis(GSEA) was carried out using differential expressed gene lists ranked by the "stat" value obtained from DESeq2 and was performed using the function "GSEA" from ClusterProfiler (v4.2.2)[14] and using the Hallmark geneset "h.all.v2023.1.Hs.symbols.gmt", downloaded from MSigDB. Significant genesets were selected with a padj < 0.05. DNMT3A transcripts level abundance reported as TPMs by rsem was used to generate **Fig. 3C**.

Quantitative Real-Time PCR Analysis

For real-time qPCR, PowerUP SYBR Green (Applied Biosystems, Foster City, CA), MicroAmp Optical 384-Well Reaction Plate (Applied Biosystems), and the Applied Biosystems QuantStudio 7 were used according to the instructions provided by the manufacturers. For the detection of different DNMT3A transcripts we used previously validated primers[15]. See primers used in the Supplementary Table 9.

Downregulation of DNMT3A expression in HUDEP-2 cells using CRISPRi

Endogenous expression of DNMT3A gene was downregulated in the cord blood CD34 cell derived erythroid cell line HUDEP-2[16,17] (a kind gift from Andre Lieber, University of Washington, Seattle, WA) using the CRISPR interference (CRISPRi) approach. A dCas9 fused with the transcriptional repressors KRAB and MeCP2 (Expression vector: pLV[Exp]-CBh>dCas9-KRAB-MeCP2:T2A:Hygro from VectorBuilder) was introduced into HUDEP-2 cells together with either a scramble sgRNA (Expression vector: pLV[gRNA]-EGFP:T2A:Neo-U6>Scramble[gRNA#1], guide sequence: *GTGTAGTTCGACCATTCGTG*, marker: EGFP:T2A:Neo from VectorBuilder] or with a gRNA targeting the *DNMT3A* promoter (Expression vector: pLV[gRNA]-TagBFP2:T2A:Neo-U6>{DNMT3A_antisense_2}, guide sequence: *ACGGTAATGAGCGCCGCTGC*, marker: TagBFP2:T2A:Neo from VectorBuilder). Detailed description of the CRISPRi approach can be found here: https://en.vectorbuilder.com/products-services/service/crispr-solutions.html. Full plasmid maps and sequences will be provided upon request. Efficient downregulation of DNMT3A expression was verified in HEK-293T, HUDEP-2 and K562 cells prior to generation of stably transduced HUDEP-2 cells. Lentiviral transduction of HUDEP-2 cells with dCas9-KRAB-MeCP2 + control (scramble) gRNA or dCas9-KRAB-MeCP2 + DNMT3A gRNA in form of lentiviral particles packaged using third generation lentiviral packaging system as described previously[18]. Regular HUDEP-2 culture media[16] was supplemented with hygromycin (100 µg/ml the first two weeks, 50 µg/ml the following six weeks due to visibly higher sensitivity of the cells during the initial culture period and 200 µg/ml for the remainder of the culture, up to seven months after transduction) starting on day 6 after transduction. Transduction efficiency was >90% at all times. Following a fluorescence-based sort of transduced cells (> 99%), purity was monitored

and RNA samples were isolated between four and seven months after transduction and subjected to RNA sequencing analysis: 25 total RNA were processed using the NEBNext® Single Cell/Low Input RNA Library Prep Kit for Illumina® (Catalog #E6420S, New England Biolabs) as per manufacturer's instructions. cDNA (20 ng input standardized for fragmentation, end repair, 5' phosphorylation and dA tailing followed by adapter ligation) and final library quality was monitored using Agilent 2100 Bioanalyzer trace analysis (Agilent Technologies). Pooled libraries were sequenced on an Illumina NovaSeq X Plus 10B (2x150) flow cell to a depth of 100 M read pairs (150 bp) per sample at Genome Technology Access Center (GTAC) and McDonnell Genome Institute at the Washington University School of Medicine. Basecalls and demultiplexing were performed with Illumina's bcl2fastq2 software. RNA-seq reads were then aligned and quantitated to the Ensembl release 101 primary assembly with an Illumina DRAGEN Bio-IT on-premise server running version 3.9.3-8 software. Gene level raw transcript counts were used for analysis of differential expression using DESeq2 (v.1.34.0) within R (version 4.1.1) programming environment. Design parameter was set on "gRNA+ Isolation time point of RNA samples". Genes were considered as differentially expressed if $p_{adj} < 0.05$. Gene Set Enrichment analysis (GSEA[19]) as well as Ingenuity Pathway Analysis (IPA, Qiagen) were carried out using differential expressed gene lists ranked by the "stat" value obtained from DESeq2 (Supplementary Table 12). GSEA was conducted using the GSEA desktop application (Broad Institute) and the hallmark gene sets as reference. IPA was conducted using the commercially available software (https://www.qiagenbioinformatics.com/products/ingenuity-pathway-analysis/, Qiagen).

In addition to whole transcriptome analysis, competitive culture experiments were performed on control gRNA (GFP+) vs. DNMT3A promoter targeting gRNA (BFP+) HUDEP-2 cells. For that, cells were cultured in regular culture media[16] without EPO for 3 days prior to setup of the coculture. Cell count based mixture of GFP and BFP + cells i.e. the input for subsequent culture in regular vs. erythroid differentiation media[16] was quantified and subsequently used as reference for relative contribution of control vs. DNMT3A downregulated HUDEP-2 cells. Co-culture composition was monitored by flow cytometry (Gallios™ flow cytometer, Beckman Coulter Life Science, Krefeld, Germany).

Single Cell DNA (scDNA) and immunophenotype analysis of primary samples

As during main screening of FD and CD cohorts, PB samples were collected in the form of buffy coats when they became available 1-3 days after whole blood donation. Since availability of the samples with specific mutations during the set collection period was random (between February 2023 and July 2023), two additional samples from *DNMT3A* R882H positive CH patients from a stroke cohort, without hematological abnormalities, were included. The full list of samples and corresponding characteristics is shown in Supplementary Table 14. A custom designed DNA sequencing panel was used for mutational analysis (Supplementary Table 15a).

A total of 74537 single cells were profiled with a genotyping efficiency for all analyzed *DNMT3A* mutations above 90 %. A total of 13 immunomagnetically CD34-enriched samples, including unmobilized peripheral blood samples from healthy blood donors and stroke patients, mobilized apheresis samples from multiple myeloma patients as well as one bone marrow sample obtained during hip replacement surgery, were processed using Protein+DNA Tapestri platform (**Supplementary Fig. 6a**) and were used to create a reference data set for human CD34+ cell enriched samples. The corresponding UMAP is shown in **Fig. 4A**.

Primary samples with known DNMT3A mutations selected for detailed analysis in the current work are listed in Supplementary table 14. Processing of the samples for scDNA seq with antibody-oligonucleotide staining was performed using the Mission Bio Tapestri scDNA-seq V2 or V3 platform as per the manufacturer's instructions. A custom scDNA-seq panel consisting of 88 amplicons targeting sample specific mutations (DNMT3A, TET2, Supplementary Table 15a) as well as amplicons that cover known high minor allele frequency SNPs from the dbSNP database (amplicons located on chromosome 10) and EPOR was designed and manufactured using Tapestri Designer (Mission Bio, San Franciso, CA) and Mission Bio Inc., respectively. A ready to use oligonucleotide-conjugated antibody (AOC) panel in form of the TotalSeq-D_Human_Heme_Oncology_Cocktail (Biolegend) was spiked with custom antibodies (in TotalSeq-D format, from Biolegend) as listed in Supplementary Table 15b. For each sample (pooled or singular analysis) approximately 1e6 cells were used as starting material for the surface staining. 35-120 e3 cells per sample were subsequently subjected to encapsulation, lysis and cell barcoding on the Tapestri instrument. Ready to sequence libraries were generated by amplifying DNA and surface protein libraries with Mission Bio V2 or V3 Index Primers. Libraries were pooled and subjected to paired-end 150-bp sequencing on a Novaseq 6000 (Illumina®, DKFZ Genomics and Proteomics Core Facility) as per manufacturer's instructions (https://support.missionbio.com/hc/en-us/articles/360046831113-Tapestri-Library-Quantification-and-Pooling-Tool). Demultiplexed, raw FASTQ files were analyzed using the Tapestri pipeline (Mission Bio). A total of 234 (#IB19), 245 (#IB45), 22196 (#BD3), 1938 (#BD43) and 1938 (#BD371) cells were recovered.

After obtaining the h5 files from the Mission Bio Pipeline, we used Mission Bio's Mosaic tool (https://github.com/MissionBio/mosaic) for extracting cells-times-surface-protein-expression matrices together with information about mutations in tabular format. All subsequent analyses were performed in R using Seurat v4.3.0(*21*). Briefly, cells with fewer than five surface proteins expressed or a total read count below 100 were removed from the dataset. Data were normalized using the centered-log-ratio (CLR) transformation. We then used Harmony(*22*) for batch correction and constructed a joint low-dimensional embedding for all of the samples together. To facilitate the annotation of cell types in the protein space, we transferred labels from a large single-cell proteo-transcriptomic atlas(*23*) using Seurat's *MapQuery* function and used the annotation to further explore surface protein expression in the low dimensional space. As a last step, we computed pseudotimes using Slingshot(*24*).

Statistical analysis

Comparisons between the frequent and control donor group with respect to the probability of observing at least one mutation from any gene at VAF threshold 0.5% or 2% were obtained as odds-ratios (OR) based on binomial generalized linear model fits. VAF scores associated with each mutation were compared after log-transformation. Differences in expected log-VAF scores between the two donor groups were tested by fitting linear mixed models, including random intercept terms for donor and gene (where relevant) grouping. All group effects were estimated while controlling for donor age and sequencing depth. Stability scores, fitness scores and site mutation rate values were also compared via robust linear modeling; fitness scores were log-transformed to improve model fitting stability. The models controlled for donor age and included random intercept terms for donor and gene (where relevant) grouping. Pooled (cohort 1 and 2) analyses additionally controlled for potential cohort effect. All continuous explanatory variables were included after standardization to enhance interpretability and model fitting stability. Analyses were run

in R software, v 4.1.1. Linear mixed model fitting was performed via maximum likelihood with the lme4 R package[20] and p-values obtained via the lmerTest R package (Satterthwaite approximation)[21]. 6776Robust linear mixed models were fitted with the robustlmm R package[22], and p-values obtained again via the Satterthwaite approximation. Finally, robust modeling without random effects was performed with robustbase R package[23]. Statistical methods used for analysis of in vitro HSPC culture results are outlined in the figure legends. Sample size was not predetermined. Data are presented as means with standard deviation (SD) to indicate the variation within each experiment. For each biological donor a paired t-test was used to compare the percentage of the *DNMT3A*-mutant clones between different conditions.

Supplementary References

1. Association, G. M. Guideline for Manufacturing of Blood and Blood Components and Hemotherapy (Hemotherapy Guidelines). *Deutscher Aerzteverlag, Cologne*.
2. Arends, C. M. *et al.* Hematopoietic lineage distribution and evolutionary dynamics of clonal hematopoiesis. *Leukemia* **32**, 1908–1919 (2018).
3. Xu, C. *et al.* smCounter2: an accurate low-frequency variant caller for targeted sequencing data with unique molecular identifiers. *Bioinformatics* **35**, 1299–1309 (2019).
4. Agrawal, M. *et al.* TET2-mutant clonal hematopoiesis and risk of gout. *Blood* **140**, 1094–1103 (2022).
5. Tate, J. G. *et al.* COSMIC: the Catalogue Of Somatic Mutations In Cancer. *Nucleic Acids Research* **47**, D941–D947 (2018).
6. Huerga Encabo, H., Ulferts, R., Sharma, A., Beale, R. & Bonnet, D. Infecting human hematopoietic stem and progenitor cells with SARS-CoV-2. *STAR Protoc* **2**, 100903 (2021).
7. Waterhouse, A. *et al.* SWISS-MODEL: homology modelling of protein structures and complexes. *Nucleic Acids Res* **46**, W296-w303 (2018).
8. Bienert, S. *et al.* The SWISS-MODEL Repository-new features and functionality. *Nucleic Acids Res* **45**, D313-d319 (2017).
9. Zhang, Z.-M. *et al.* Structural basis for DNMT3A-mediated de novo DNA methylation. *Nature* **554**, 387–391 (2018).
10. Pettersen, E. F. *et al.* UCSF ChimeraX: Structure visualization for researchers, educators, and developers. *Protein Sci* **30**, 70–82 (2021).
11. Schroedinger DeLano W., L. PyMol. Retrieved from http://www.pymol.org.pymol. (2020).
12. Dobin, A. *et al.* STAR: ultrafast universal RNA-seq aligner. *Bioinformatics (Oxford, England)* **29**, 15–21 (2013).
13. Li, B. & Dewey, C. N. RSEM: accurate transcript quantification from RNA-Seq data with or without a reference genome. *BMC bioinformatics* **12**, 323 (2011).
14. Wu, T. *et al.* clusterProfiler 4.0: A universal enrichment tool for interpreting omics data. *Innovation (Cambridge (Mass.))* **2**, 100141 (2021).
15. Božić, T. *et al.* Variants of DNMT3A cause transcript-specific DNA methylation patterns and affect hematopoiesis. *Life science alliance* **1**, e201800153 (2018).
16. Li, C. *et al.* In Vivo HSC Gene Therapy Using a Bi-modular HDAd5/35++ Vector Cures Sickle Cell Disease in a Mouse Model. *Mol Ther* **29**, 822–837 (2021).
17. Kurita, R. *et al.* Establishment of immortalized human erythroid progenitor cell lines able to produce enucleated red blood cells. *PLoS One* **8**, e59890 (2013).
18. Xiang, J. *et al.* An 'off-the-shelf' CD2 universal CAR-T therapy for T-cell malignancies. *Leukemia* **37**, 2448–2456 (2023).

19. Subramanian, A. *et al.* Gene set enrichment analysis: a knowledge-based approach for interpreting genome-wide expression profiles. *Proceedings of the National Academy of Sciences of the United States of America* **102**, 15545–15550 (2005).
20. Bates, D., Mächler, M., Bolker, B. & Walker, S. Fitting Linear Mixed-Effects Models Using lme4. *Journal of Statistical Software* **67**, 1–48 (2015).
21. Kuznetsova, A., Brockhoff, P. B. & Christensen, R. H. B. lmerTest Package: Tests in Linear Mixed Effects Models. *Journal of Statistical Software* **82**, 1–26 (2017).
22. Koller, M. robustlmm: An R Package for Robust Estimation of Linear Mixed-Effects Models. *Journal of Statistical Software* **75**, 1–24 (2016).
23. Maechler M RP, Croux C, Todorov V, Ruckstuhl A, Salibian-Barrera M, Verbeke T, Koller M, Conceicao EL, A. di P. M. robustbase: Basic Robust Statistics. Preprint at (2022).
24. Watson, C. J. *et al.* The evolutionary dynamics and fitness landscape of clonal hematopoiesis. *Science (1979)* **367**, 1449–1454 (2020).

Supplementary Figure Legends

Fig. S1: Clonal landscape in blood donors

(**A**) VAF of all CH variants in the FD vs. CD detected at VAF ≥0.005. n.s.: not significant. (**B**) Number of donors with CH at VAF ≥0.005 with the indicated number of mutations detected per donor. Data from the cohort 1 and 2 are plotted together.

(**C**) All variants observed at a VAF ≥ 0.005, in CD (blue) and FD (red). Genes were ordered by the number of observed hits. For clarity, genes in which a mutation was found only once are indicated as *. These are *ABL1, ANKRD26, ASXL2, ATM, BCR, BRCA1, C17orf97, CBL, DDX41, DNMT1, ETV6, FIP1L1, GJB3, IL7R, KCNK13, KLHL6, LUC7L2, MAP2K1, MSH6, NBN, PDGFRA, PRPF8, RAD21, RELN, RUNX1, SETBP1, SF3A1, SH2B3, SMC3, TAL1, TP53, U2AF1* and *WAS* in CD cohort and *ASXL2, ATM, BCOR, BLM, BRCA1, BRCA2, BRINP3, CHEK2, CREBBP, CRLF2, CSF3R, CTCF, CUX1, DNMT1, EGFR, EP300, FAM154B, FAM47A, FBXW7, GJB3, GNAS, HNRNPK, IDH1, KAT6A, KCNK13, KMT2A, LRRC4, MYD88, NSD1, NTRK3, PML, PRF1, PRPF40B, RAD21, RELN, RP11-816J6.3, RUNX1, SETBP1, SMC1A, SRSF2, TAL1, WAS, WRN* and *XPO1* in FD cohort. Data from cohort 1 and 2 are plotted together. (**D**) VAF of all *DNMT3A* variants in the frequent vs. control donor cohorts detected at a VAF ≥ 0.005. Data from cohort 1 and 2 are plotted together. (**E**) Structural models of the *DNMT3A* variants W305*, S663fs (704*) and E733* in direct comparison to the WT protein were generated using homology modeling on SWISS-MODEL[83,84] based on the crystal structure of DNMT3A available on PDB under the alias 5YX2[37]. (**F**) Analysis of the site-specific mutation rate for DNMT3A mutations from the frequent and control donor cohort that were matched to the variants characterized by Watson *et al.* [24] (See Supplementary Table 5). Data from cohort 1 and 2 cohorts are plotted together. (**G-H**) Longitudinal analysis of variants. Samples were collected at a second timepoint (approximately 1 year later, see Supplementary Tables 6 and 7 for details). Six *DNMT3A* variants from the CD cohorts **(G)** and 16 *DNMT3A* variants from the FD **(H)** were analyzed. A VAF of 0.000 for the second donation of Donor #444 indicates that the mutant allele was not detected at the set VAF cutoff of 0.005. However, a VAF of 0.002 was determined using the actual read count ratio in this sample. Variants that showed a change in the VAF between the two time points are highlighted in the legend on the right.

Fig. S2. Mutations in frequent blood donors are found in myeloid and lymphoid cell lineages.

(**A**) Schematic presentation of analysis of mature and immature cell fractions isolated using FACS from PBMC of the frequent blood donors Donor 8, 31, 42 and the control blood donor 371. Question mark indicates fraction sorted directly. Question mark in brackets indicates projected presence of a tested mutation. (**B**) VAF of each mutation was analyzed using digital droplet PCR (ddPCR) performed concurrently on the whole PBMC samples (timepoint 1 and 2) as well as the CD34+ cells, T- and B-cells and monocytes collected at the second donation timepoint. All five variants tested were detected in all 4 sorted cell populations, indicative of their presence in the myeloid (CMP, GMP) and lymphoid (CLP) lineage.

Fig. S3. EPO expands *DNMT3A*-mutant clones found in frequent blood donors.

(**A**) Sanger sequencing performed in colony forming units (CFU) to detect the presence of each *DNMT3A* mutation in one or both alleles. For each mutation a minimum of 20 colonies were picked and sequenced. (**B**) Representative flow cytometry analysis after 4 weeks in the presence of EPO or LPS. (**C**) Quantification of the myeloid ($CD33^+$) and erythroid ($CD235a^+CD71^+$) lineages found in the different conditions. (**D-E**) VAF detected in different conditions after 4 weeks in culture. Significant increase between non-treated (CTRL) and EPO conditions (3U/mL and 15U/mL) (**D**) but no differences upon LPS treatment (**E**) were detected. Each dot represents an independent biological donor. Paired t-test for each biological donor was used for

the statistical significance of the percentage of the *DNMT3A*-mutant clones between different conditions. *, $p < 0.05$. (**F**) Fold-change expansion (treated with EPO or IFNγ, compared to untreated condition) of different mutations introduced in bone marrow HSPCs (4-6 biological donors tested). Each dot represents an independent biological donor. For each biological donor, a paired t-test was used to compare the percentage of the *DNMT3A*-mutant clones between different conditions. (**G**) Co-culture of *DNMT3A* mutations, found in FD cohort in presence of preleukemic R882 variants. Each dot represents an independent biological donor. t-test for each biological donor between different conditions was used for the statistical significance of the percentage of the *DNMT3A*-mutant clones.

Fig. S4. Characterization of *DNMT3A* FD mutation W305* in K562 clones.
(**A**) Pathway enrichment analysis reveal that W305* mutation specifically causes an upregulation of transcriptional programs associated with heme metabolism in K562 cells. (**B**) Volcano plot showing the genes specifically upregulated (red) or downregulated (blue) in W305* mutant K562. See Supplementary Table 11 for a detailed list of genes. (**C**) RT-qPCR based analysis of *DNMT3A* transcript 1, 2 and 4 expression in K562 clones. W305* K562 clones show lower expression of Transcript 2 while R882 variants express higher levels of this *DNMT3A* transcript. Each dot represents an independent experiment. t-test was used for statistical significance. *, $p < 0.05$; **, $p < 0.01$; *** $p < 0.001$.

Fig. S5. Characterization of *DNMT3A* downregulation in HUDEP-2 cells.
(**A**) Schematic representation of the CRISPR interference (CRISPRi) mediated downregulation of *DNMT3A* expression in HUDEP-2 cells followed by transcriptional and functional analysis. (**B**) Normalized *DNMT3A* counts (DESeq2) in *DNMT3A* downregulated vs. control HUDEP-2 cells. n=9. (**C**) Gene set enrichment analysis (GSEA) of IFNγ and IFNα response signature and allograft rejection associated genes in *DNMT3A* downregulated vs. control HUDEP-2 cells. ES, enrichment score; NES, normalized enrichment score; FDR, false discovery rate. (**D**) Ingenuity Pathway Analysis (IPA) of HUDEP-2 cells (*DNMT3A* downregulated vs. control). Positive (orange), negative (blue) activity patterns are presented as well as significantly associated pathways with no activity pattern (gray). (**E**) *DNMT3A* downregulated (BFP+) and HUDEP-2 control (GFP+) cells were co-cultured at the given ratios in regular and erythroid differentiation media. The ratio between BFP and GFP positive cells was analyzed over a period of 8 days and is presented relative to the input. n=3, three independent experiments, measurement in duplicates. P-values Differentiation vs. Regular Media for 1:10 culture at timepoints Day 1, 3, 5 and 8 were: 0.29, 0.20, 0.06 and 0.06, respectively. P-values Differentiation vs. Regular Media for 1:1 culture at timepoints Day 1, 3, 5 and 8 were: 0.41, 0.27, 0.13 and 0.06, respectively.

Fig. S6. R882 associated myeloid bias is not found in hematopoiesis derived from HSCs carrying FD enriched *DNMT3A* mutations.
(**A**) Schematic representation of single cell DNA and surface marker expression analysis using Tapestri DNA+Protein platform. (**B-E**) Intradonor/Intrapatient, genotype specific cellular composition of indicated donor samples. 15 cell clusters were defined according to the UMAP in Fig. 4A as shown in the color-matched legend. Data is also presented as bar plots to help visualize the difference in contribution of R882 mutant vs. non-mutant cells within the 15 cell clusters. Fisher exact test was used for analysis of statistical significance in the contribution of a mutant vs. non-mutant genotype to a given cell population *, $p < 0.05$; **, $p < 0.01$; *** $p < 0.001$.

Fig. S7. R882 mutant hematopoiesis is associated with a myeloid bias both in primary samples and a humanized mouse model.

(**A**) Contribution of mutant vs. non-mutant cells within the lymphoid (B cells, CD4+ T cells, CD8+ T cells, NK cells) and myeloid (classical monocytes, non-classical monocytes, dendritic cells (DCs)) fraction for the indicated donor samples. (**B**) Changes in lineage contribution for mutant vs. non-mutant genotype found to be common between all non-EPO responsive *DNMT3A* mutations listed on the left. (**C**) Representative flow cytometry gating strategy to sort each cell population in humanized mice engrafted with human HSCs carrying W305* or R882H after sustained erythropoietic stress to mimic the environmental pressure of the FD cohort.

Figure S1

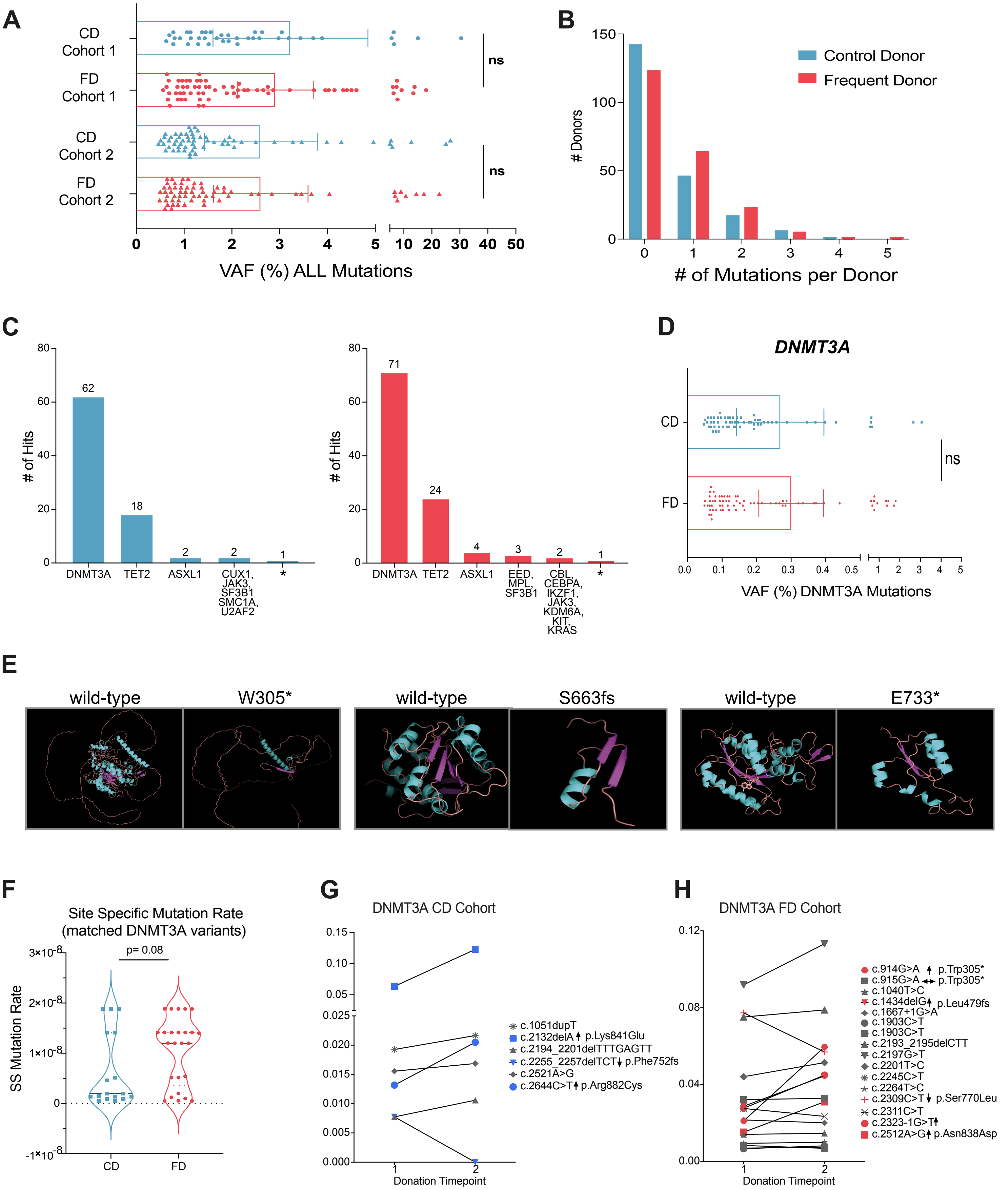

A
CD Cohort 1
FD Cohort 1
CD Cohort 2
FD Cohort 2
ns
ns
0 1 2 3 4 5 10 20 30 40 50
VAF (%) ALL Mutations
B
150
100
50
0
Donors
Control Donor
Frequent Donor
0 1 2 3 4 5
# of Mutations per Donor
C
80
60
40
20
0
# of Hits
62
18
2
2
1
DNMT3A
TET2
ASXL1
CUX1, JAK3, SF3B1 SMC1A, U2AF2
*
71
24
4
3
2
1
DNMT3A
TET2
ASXL1
EED, MPL, SF3B1
CBL, CEBPA, IKZF1, JAK3, KDM6A, KIT, KRAS
*
D
DNMT3A
CD
FD
ns
0.0 0.1 0.2 0.3 0.4 0.5 1 2 3 4 5
VAF (%) DNMT3A Mutations
E
wild-type
W305*
wild-type
S663fs
wild-type
E733*
F
Site Specific Mutation Rate (matched DNMT3A variants)
p= 0.08
SS Mutation Rate
CD
FD
G
DNMT3A CD Cohort
Donation Timepoint
c.1051dupT
c.2132delA p.Lys841Glu
c.2194_2201delTTTGAGTT
c.2255_2257delTCT p.Phe752fs
c.2521A>G
c.2644C>T p.Arg882Cys
H
DNMT3A FD Cohort
Donation Timepoint
c.914G>A p.Trp305*
c.915G>A p.Trp305*
c.1040T>C
c.1434delG p.Leu479fs
c.1667+1G>A
c.1903C>T
c.1903C>T
c.2193_2195delCTT
c.2197G>T
c.2201T>C
c.2245C>T
c.2264T>C
c.2309C>T p.Ser770Leu
c.2311C>T
c.2323-1G>T
c.2512A>G p.Asn838Asp

Figure S2

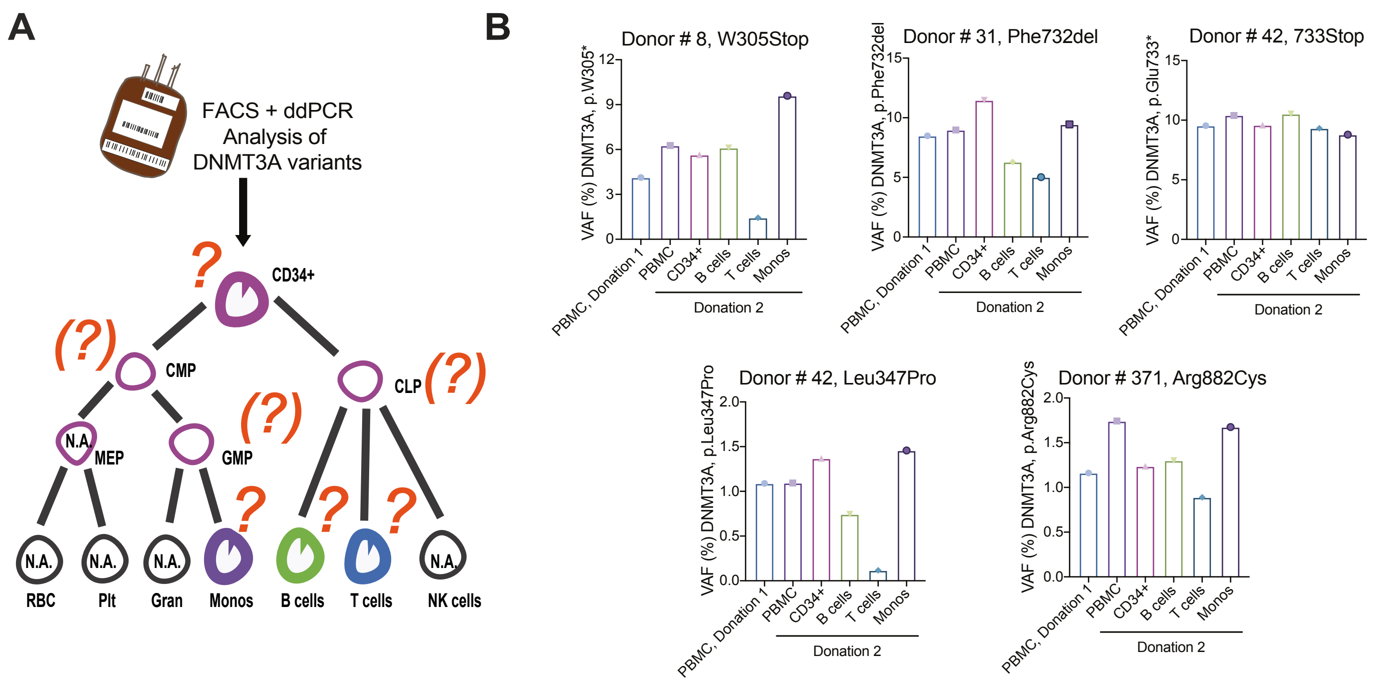
A
FACS + ddPCR
Analysis of
DNMT3A variants
CD34+
CMP
CLP
MEP
GMP
N.A.
RBC
Plt
Gran
Monos
B cells
T cells
NK cells
B
Donor # 8, W305Stop
VAF (%) DNMT3A, p.W305*
Donor # 31, Phe732del
VAF (%) DNMT3A, p.Phe732del
Donor # 42, 733Stop
VAF (%) DNMT3A, p.Glu733*
Donor # 42, Leu347Pro
VAF (%) DNMT3A, p.Leu347Pro
Donor # 371, Arg882Cys
VAF (%) DNMT3A, p.Arg882Cys
PBMC, Donation 1
PBMC
CD34+
B cells
T cells
Monos
Donation 2

Figure S3

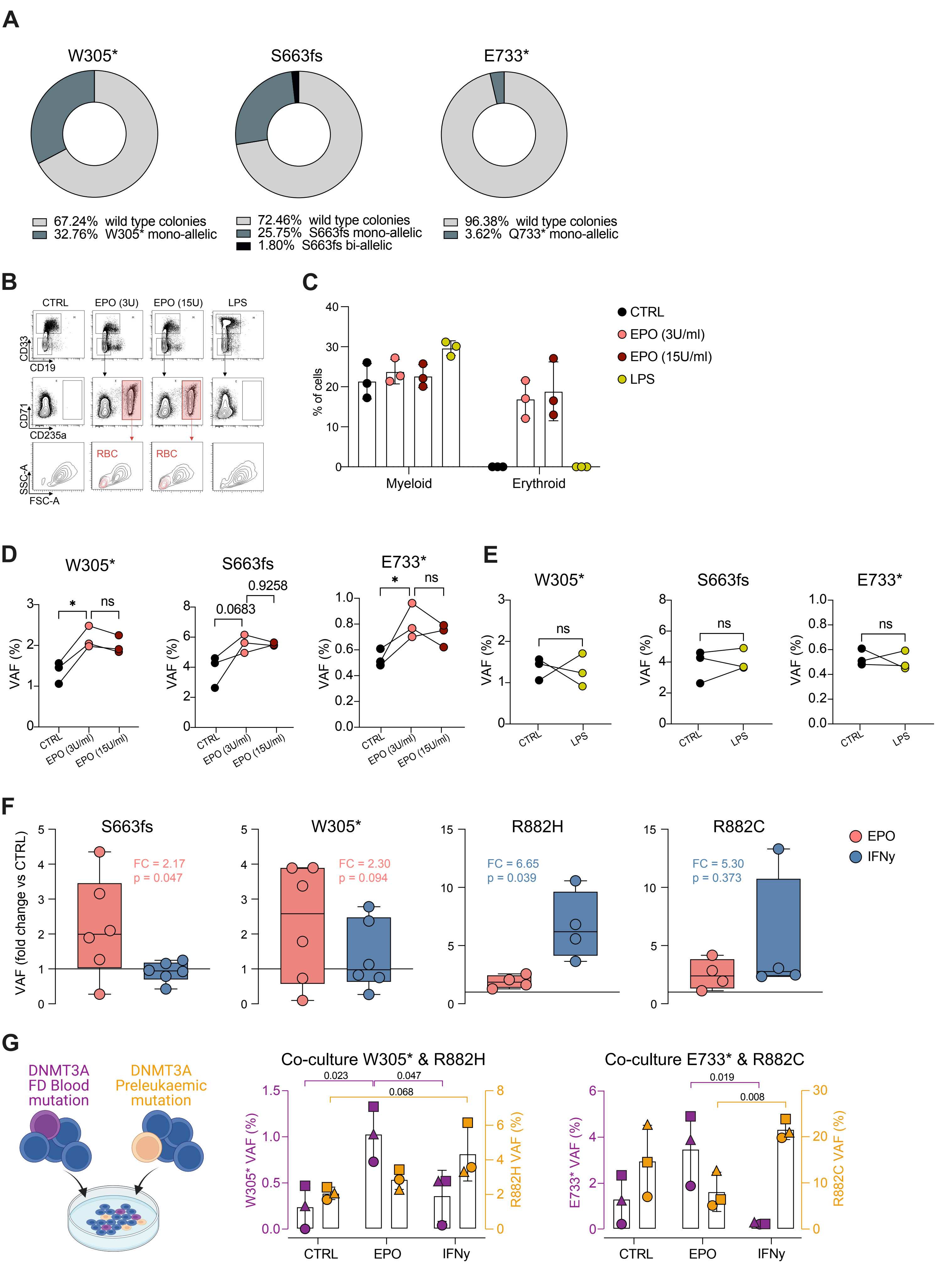

A
W305*
S663fs
E733*
67.24% wild type colonies
32.76% W305* mono-allelic
72.46% wild type colonies
25.75% S663fs mono-allelic
1.80% S663fs bi-allelic
96.38% wild type colonies
3.62% Q733* mono-allelic
B
CTRL
EPO (3U)
EPO (15U)
LPS
CD33
CD19
CD71
CD235a
RBC
SSC-A
FSC-A
C
% of cells
Myeloid
Erythroid
CTRL
EPO (3U/ml)
EPO (15U/ml)
LPS
D
W305*
S663fs
E733*
VAF (%)
0.0683
0.9258
ns
EPO (3U/ml)
EPO (15U/ml)
E
F
VAF (fold change vs CTRL)
R882H
R882C
FC = 2.17
p = 0.047
FC = 2.30
p = 0.094
FC = 6.65
p = 0.039
FC = 5.30
p = 0.373
EPO
IFNy
G
DNMT3A
FD Blood
mutation
DNMT3A
Preleukaemic
mutation
Co-culture W305* & R882H
Co-culture E733* & R882C
0.023
0.047
0.068
0.019
0.008
W305* VAF (%)
R882H VAF (%)
E733* VAF (%)
R882C VAF (%)

Figure S4

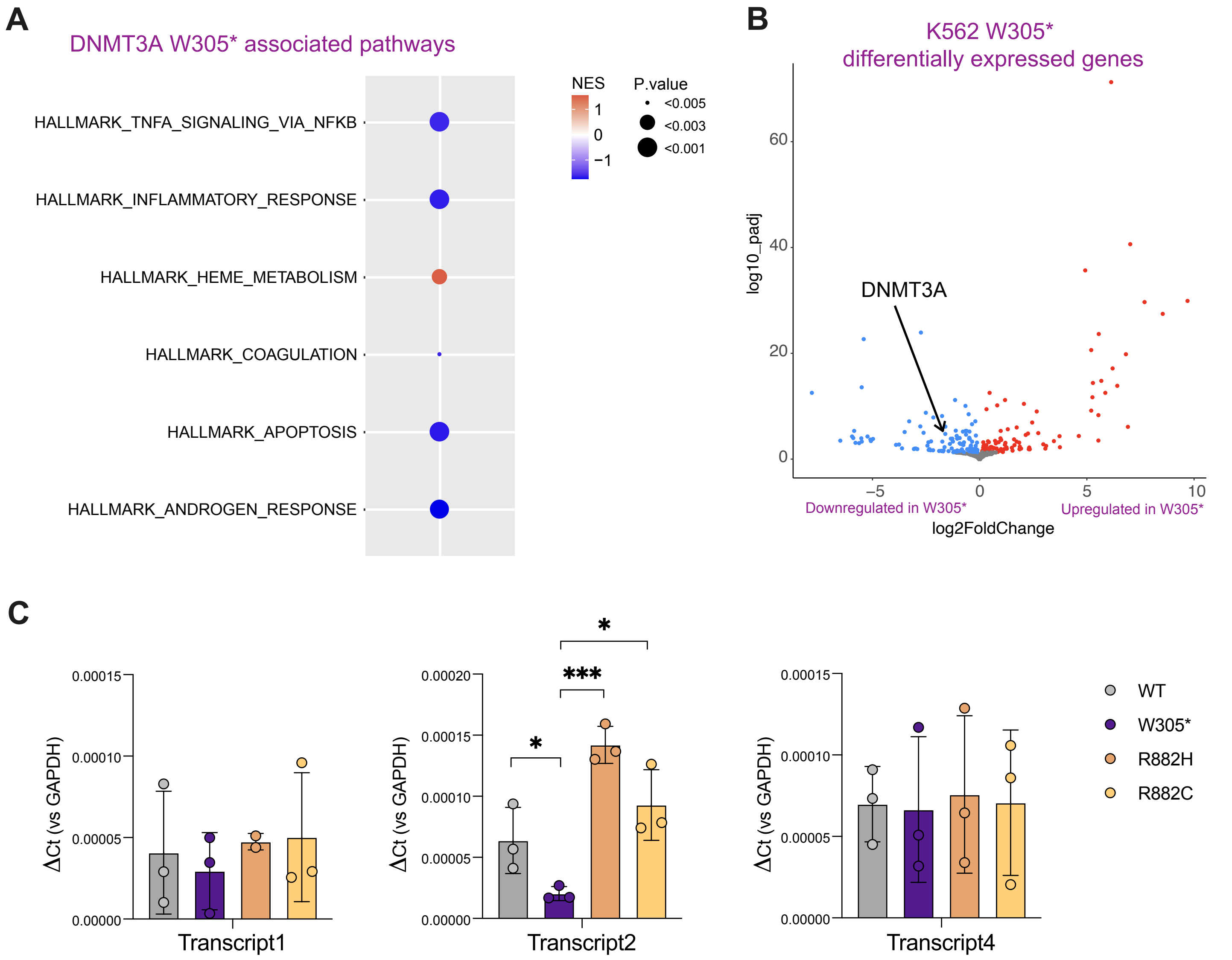

A
DNMT3A W305* associated pathways
HALLMARK_TNFA_SIGNALING_VIA_NFKB
HALLMARK_INFLAMMATORY_RESPONSE
HALLMARK_HEME_METABOLISM
HALLMARK_COAGULATION
HALLMARK_APOPTOSIS
HALLMARK_ANDROGEN_RESPONSE
NES
1
0
−1
P.value
<0.005
<0.003
<0.001
B
K562 W305*
differentially expressed genes
log10_padj
60
40
20
0
DNMT3A
−5
0
5
10
Downregulated in W305*
Upregulated in W305*
log2FoldChange
C
ΔCt (vs GAPDH)
0.00015
0.00010
0.00005
0.00000
Transcript1
0.00020
0.00015
0.00010
0.00005
0.00000
*
***
*
Transcript2
Transcript4
WT
W305*
R882H
R882C

Figure S5

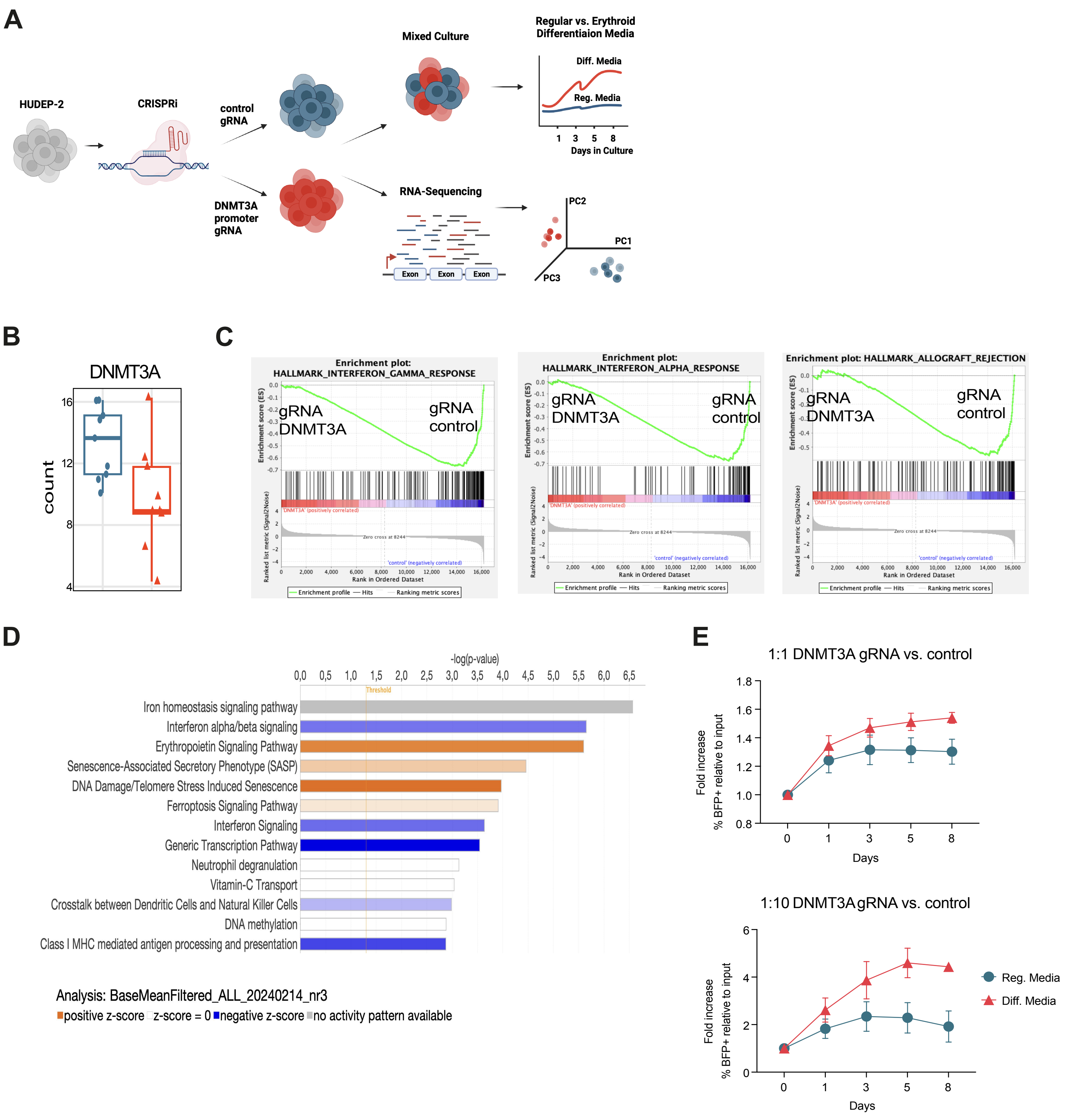
A
HUDEP-2
CRISPRi
control gRNA
DNMT3A promoter gRNA
Mixed Culture
Regular vs. Erythroid Differentiaion Media
Diff. Media
Reg. Media
1 3 5 8
Days in Culture
RNA-Sequencing
Exon
Exon
Exon
PC2
PC1
PC3
B
DNMT3A
count
C
Enrichment plot: HALLMARK_INTERFERON_GAMMA_RESPONSE
Enrichment plot: HALLMARK_INTERFERON_ALPHA_RESPONSE
Enrichment plot: HALLMARK_ALLOGRAFT_REJECTION
gRNA DNMT3A
gRNA control
Enrichment score (ES)
Ranked list metric (Signal2Noise)
Rank in Ordered Dataset
Zero cross at 8244
Enrichment profile
Hits
Ranking metric scores
D
-log(p-value)
Threshold
Iron homeostasis signaling pathway
Interferon alpha/beta signaling
Erythropoietin Signaling Pathway
Senescence-Associated Secretory Phenotype (SASP)
DNA Damage/Telomere Stress Induced Senescence
Ferroptosis Signaling Pathway
Interferon Signaling
Generic Transcription Pathway
Neutrophil degranulation
Vitamin-C Transport
Crosstalk between Dendritic Cells and Natural Killer Cells
DNA methylation
Class I MHC mediated antigen processing and presentation
Analysis: BaseMeanFiltered_ALL_20240214_nr3
positive z-score
z-score = 0
negative z-score
no activity pattern available
E
1:1 DNMT3A gRNA vs. control
1:10 DNMT3A gRNA vs. control
Fold increase % BFP+ relative to input
Days
Reg. Media
Diff. Media

Figure S6

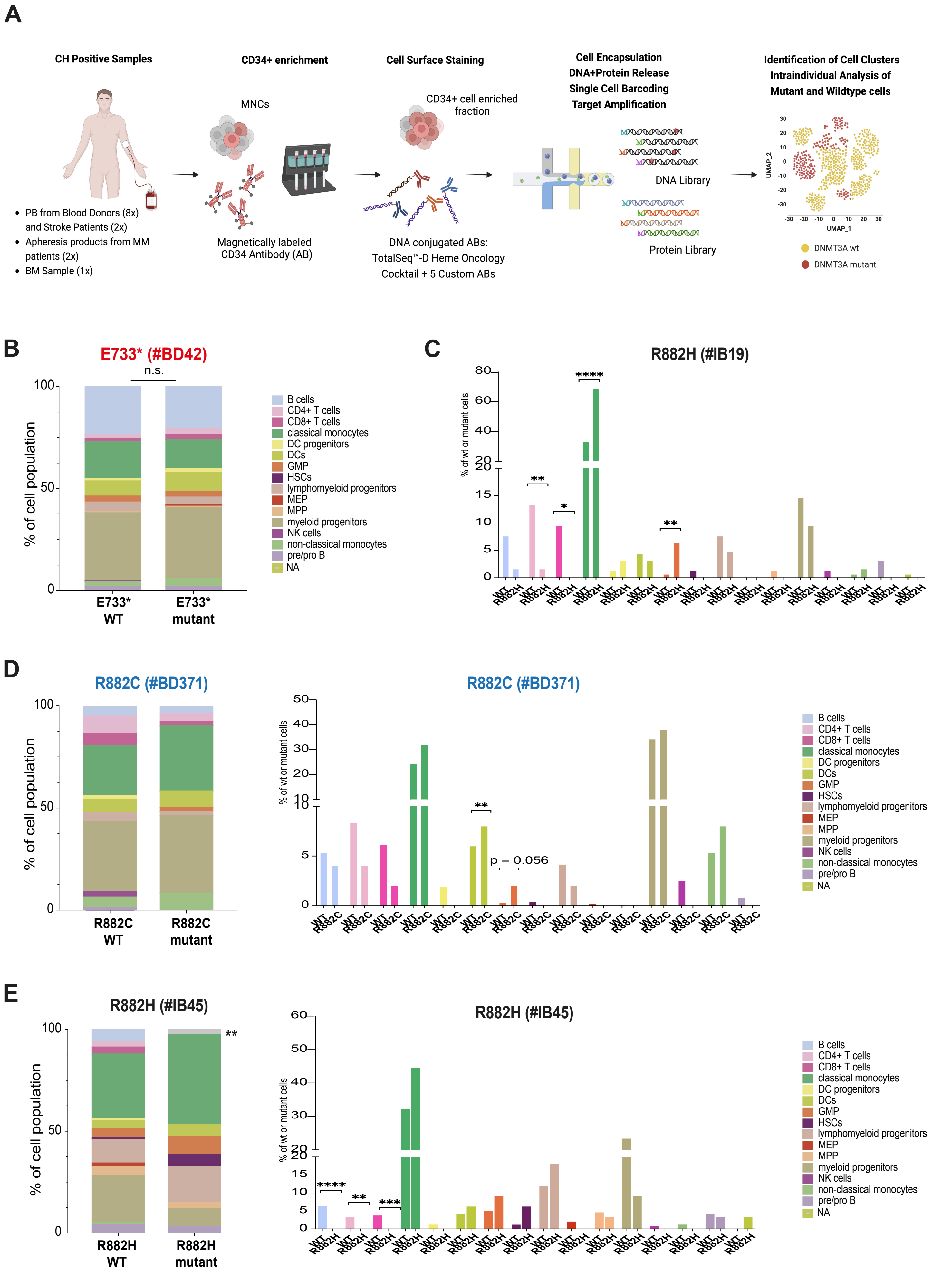
A
CH Positive Samples
PB from Blood Donors (8x) and Stroke Patients (2x)
Apheresis products from MM patients (2x)
BM Sample (1x)
CD34+ enrichment
MNCs
Magnetically labeled CD34 Antibody (AB)
Cell Surface Staining
CD34+ cell enriched fraction
DNA conjugated ABs: TotalSeq™-D Heme Oncology Cocktail + 5 Custom ABs
Cell Encapsulation
DNA+Protein Release
Single Cell Barcoding
Target Amplification
DNA Library
Protein Library
Identification of Cell Clusters
Intraindividual Analysis of Mutant and Wildtype cells
UMAP_1
UMAP_2
DNMT3A wt
DNMT3A mutant
B
E733* (#BD42)
n.s.
% of cell population
E733* WT
E733* mutant
B cells
CD4+ T cells
CD8+ T cells
classical monocytes
DC progenitors
DCs
GMP
HSCs
lymphomyeloid progenitors
MEP
MPP
myeloid progenitors
NK cells
non-classical monocytes
pre/pro B
NA
C
R882H (#IB19)
% of wt or mutant cells
****
**
*
**
D
R882C (#BD371)
% of cell population
R882C WT
R882C mutant
R882C (#BD371)
% of wt or mutant cells
**
p = 0.056
E
R882H (#IB45)
% of cell population
**
R882H WT
R882H mutant
R882H (#IB45)
% of wt or mutant cells
****
**
***

Figure S7

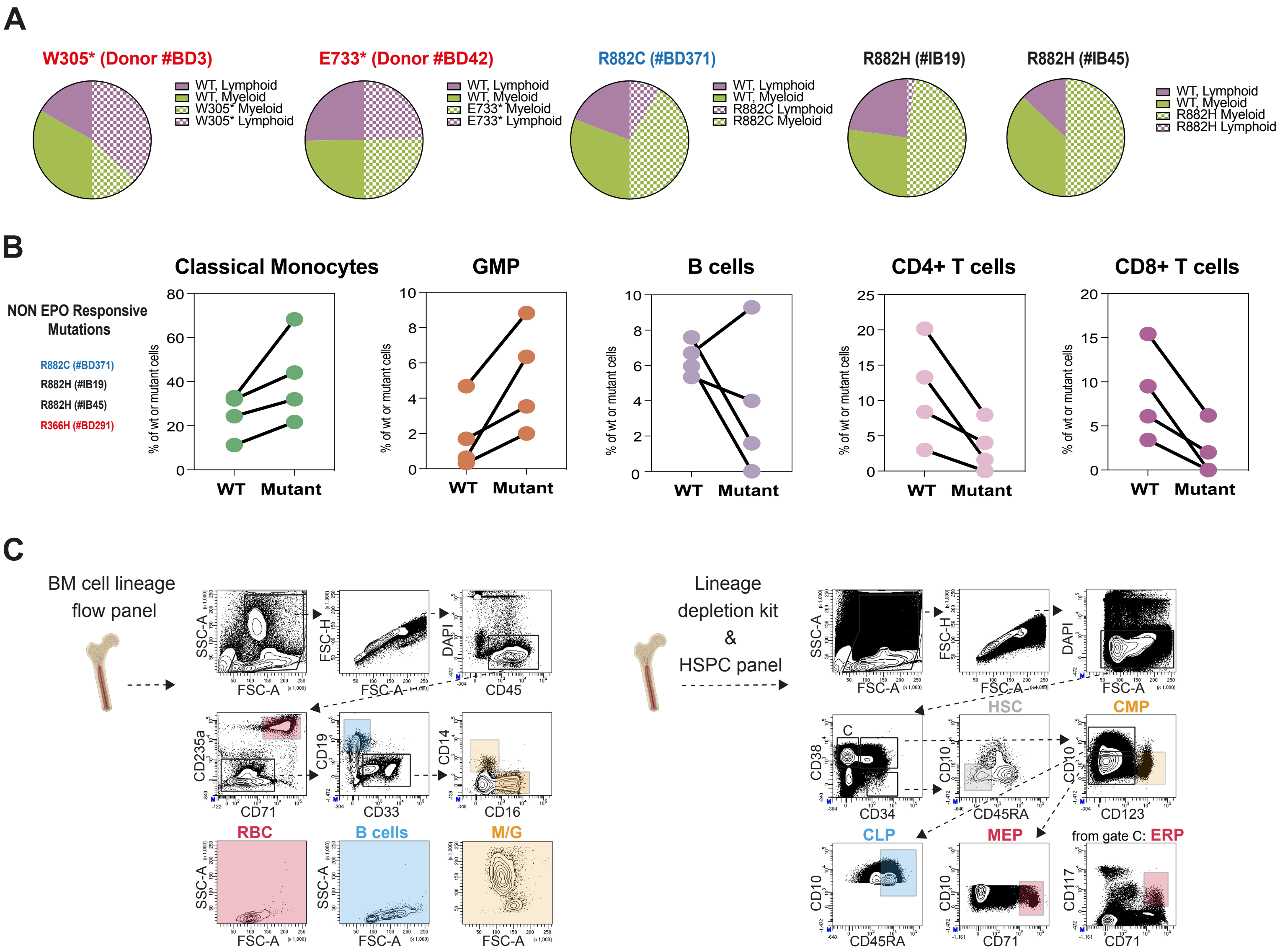
A
W305* (Donor #BD3)
WT, Lymphoid
WT, Myeloid
W305* Myeloid
W305* Lymphoid
E733* (Donor #BD42)
E733* Myeloid
E733* Lymphoid
R882C (#BD371)
R882C Lymphoid
R882C Myeloid
R882H (#IB19)
R882H (#IB45)
R882H Myeloid
R882H Lymphoid
B
NON EPO Responsive Mutations
R882C (#BD371)
R882H (#IB19)
R882H (#IB45)
R366H (#BD291)
Classical Monocytes
GMP
B cells
CD4+ T cells
CD8+ T cells
% of wt or mutant cells
WT
Mutant
C
BM cell lineage flow panel
Lineage depletion kit & HSPC panel
SSC-A
FSC-A
FSC-H
DAPI
CD45
CD235a
CD71
CD19
CD33
CD14
CD16
RBC
B cells
M/G
CD38
CD34
CD10
CD45RA
CD123
HSC
CMP
CLP
MEP
from gate C: ERP
CD117
